# The role of the two-component systems Cpx and Arc in protein alterations upon gentamicin treatment in *Escherichia coli*

**DOI:** 10.1186/s12866-017-1100-9

**Published:** 2017-09-18

**Authors:** Emina Ćudić, Kristin Surmann, Gianna Panasia, Elke Hammer, Sabine Hunke

**Affiliations:** 1 FB 5 Microbiology, Department of Biology/Chemistry, University Osnabrück, Barbarastraße 11, 49076 Osnabrück, Germany; 2 Department of Functional Genomics, Interfaculty Institute of Genetics and Functional Genomics, University Medicine Greifswald, Friedrich-Ludwig-Jahn-Straße 15A, 17475 Greifswald, Germany; 3 Department of Biology, Institute of Molecular Microbiology and Biotechnology, Universität Münster, Corrensstraße 3, 48149 Münster, Germany

**Keywords:** *E. coli*, Two - component system, Cpx, Arc, Aminoglycosides, Protein interactions, Absolute quantification, SRM, Proteome profiling

## Abstract

**Background:**

The aminoglycoside antibiotic gentamicin was supposed to induce a crosstalk between the Cpx- and the Arc-two-component systems (TCS). Here, we investigated the physical interaction of the respective TCS components and compared the results with their respective gene expression and protein abundance. The findings were interpreted in relation to the global proteome profile upon gentamicin treatment.

**Results:**

We observed specific interaction between CpxA and ArcA upon treatment with the aminoglycoside gentamicin using Membrane-Strep-tagged protein interaction experiments (mSPINE). This interaction was neither accompanied by detectable phosphorylation of ArcA nor by activation of the Arc system via CpxA. Furthermore, no changes in absolute amounts of the Cpx- and Arc-TCS could be determined with the sensitive single reaction monitoring (SRM) in presence of gentamicin. Nevertheless, upon applying shotgun mass spectrometry analysis after treatment with gentamicin, we observed a reduction of ArcA ~ P-dependent protein synthesis and a significant Cpx-dependent alteration in the global proteome profile of *E. coli*.

**Conclusions:**

This study points to the importance of the Cpx-TCS within the complex regulatory network in the *E. coli* response to aminoglycoside-caused stress.

**Electronic supplementary material:**

The online version of this article (10.1186/s12866-017-1100-9) contains supplementary material, which is available to authorized users.

## Background

Aminoglycoside antibiotics are toxic to bacterial cells by targeting the 30S subunit of the ribosome and by subsequent inhibition of the translation process [1]. However, treatment with aminoglycosides is commonly used to understand cellular processes that enable bacteria to resist these antibiotics. It was discovered that sub-inhibitory concentrations (1 μg ml^−1^) of the aminoglycoside gentamicin induce the biofilm formation of *Escherichia coli* (*E. coli*) and *Pseudomonas aeruginosa* (*P. aeruginosa*) causing aminoglycoside-resistance of these strains [2]. Both, biofilm formation and two-component system (TCS) signaling were found to increase the resistance towards aminoglycosides [2, 3]. TCSs comprise a sensor kinase (SK) and a response regulator (RR) [4–6]. After sensing a specific stimulus, the SK autophosphorylates and transfers the phosphoryl group to the respective RR. The response is terminated by dephosphorylation of the RR either by the SK if it possesses phosphatase activity, or by intrinsic activity of the RR [6, 7]. Mahoney and Silhavy (2013) found that hyper-activation of the Cpx (conjugative pilus expression)-TCS pathway in *E. coli* enhances the viability of cells grown in presence of gentamicin [3]. In contrast to this, Kohanski et al. (2008) hypothesized that treatment of *E. coli* cells with gentamicin induces a crosstalk between the Cpx-TCS and the Arc (anoxic redox control)-TCS of *E. coli* resulting in bacterial cell death [8]. This hypothesis was therefore particularly significant, as crosstalk between different TCSs is commonly circumvented by distinct mechanisms to ensure specific responses [9]. Nevertheless, TCSs are able to crosstalk under certain environmental conditions allowing for a diversified response by integration of multiple signals [10]. Crosstalk can occur by different mechanisms, such as: i) integration of additional signals by an accessory protein regulated by another TCS, ii) phosphorylation of another, non-cognate RR by the SK, and iii) target promotor recognition by a non-cognate RR [11]. Here, we refer to ‘crosstalk’ at RR-phosphorylation-level. Such crosstalk between TCS in *E. coli* has been already reported between the Cpx-TCS and the EnvZ-OmpR-TCS [12, 13], or the quorum-sensing QseBC-TCS [14].

A functional interaction between the Cpx- and Arc-TCS has been in discussion since 1980. Mutations in *cpxA* and *arcA* resulted in reduced synthesis of isoleucine and valine [15], reduced conjugation [16], and reduced synthesis of porins [17]. That time, the response regulator of the Arc-TCS, ArcA, was named CpxB as the supposed cognate response regulator of CpxA [18]. Later, the genuine cognate response regulator of CpxA, CpxR, was identified [19]. Further studies identified ArcB as the specific SK of ArcA [20, 21].

Today it is known that the Cpx-TCS consists of the SK CpxA, the RR CpxR, and the periplasmic accessory molecule CpxP [22]. The SK CpxA exhibits kinase and phosphatase activity. Its autophosphorylation is inhibited by direct interaction with a CpxP homodimer under non-activating conditions [23, 24]. The Cpx-TCS is responsible for detection and response to conditions disturbing the integrity of the cell envelope which includes a large variety of signals, e.g. elevated pH [25, 26], or changes in the membrane composition [27]. Moreover, defective lipopolysaccharide (LPS) synthesis and assembly were found to induce the Cpx-TCS [28, 29]. Activation of the Cpx-TCS results in positive or negative regulation of several genes on the transcript level [30–33] and is associated with alterations of protein abundance [34]. Additionally, the Cpx-TCS can be regulated independently of CpxP [35], for example by the outer membrane lipoprotein NlpE which is known to specifically induce Cpx-TCS activity upon surface attachment or upon its overexpression [36–38].

The Arc-TCS monitors changes during respiratory growth. ArcB is autophosphorylated under reducing conditions followed by transphosphorylation of the RR ArcA by a 3-step phosphorelay reaction in order to induce the expression of genes e.g. involved in fermentative metabolism [39–44].

**Fig. 1:**
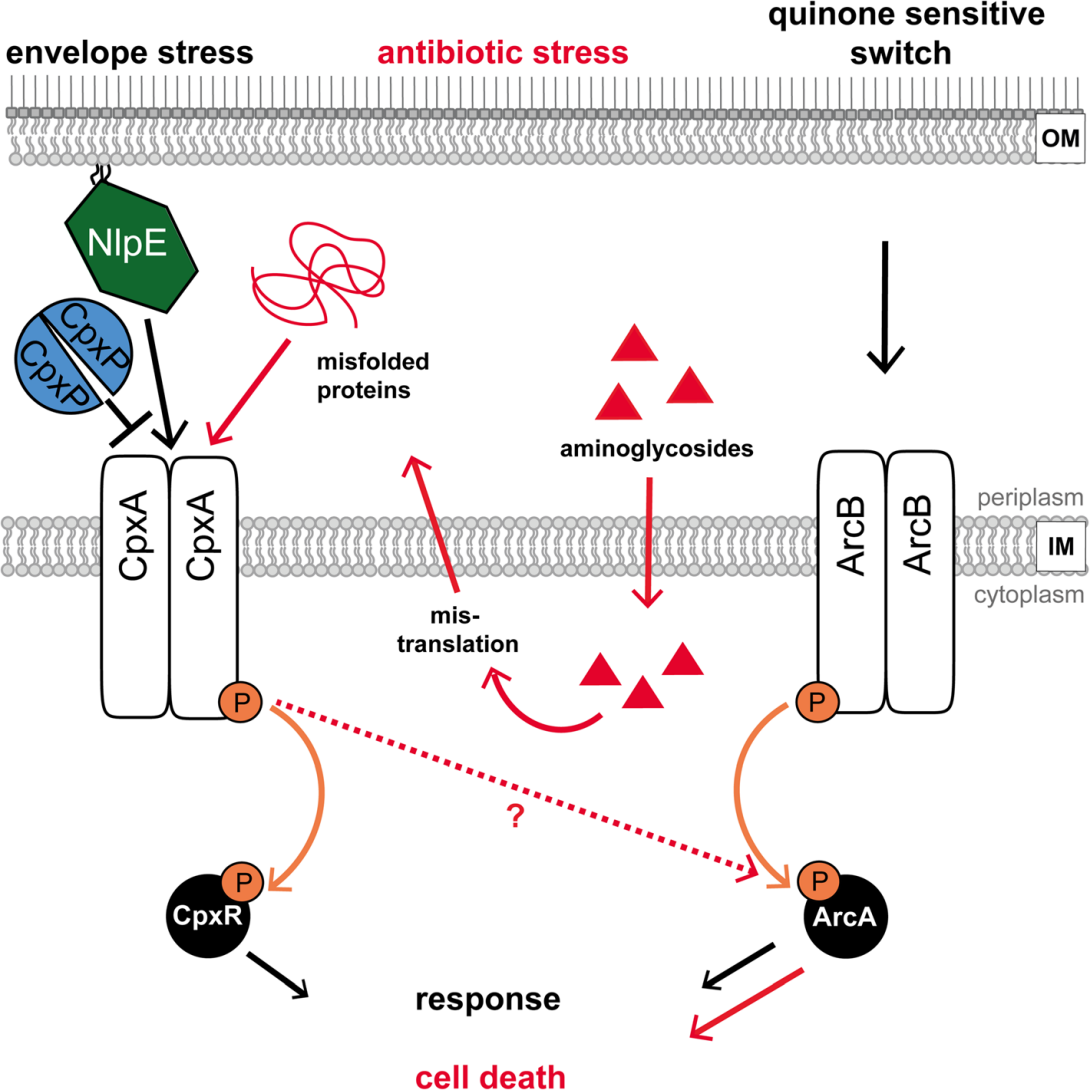
Organization and regulation of the Arc- and Cpx-TCS in *E. coli*. The Arc system is commonly induced under conditions provoking a reduced state of the quinone pool of the electron transport chain. The autophosphorylation of the SK ArcB is followed by a phosphoryl group transfer onto its cognate RR ArcA resulting in a specific response. The phosphorelay reaction for the phosphoryl group transfer from ArcB to ArcA is simplified for better understanding. The envelope stress sensing Cpx system is induced by e.g. misfolded proteins or *nlpE* overexpression, followed by autophosphorylation of CpxA, phosphotransfer onto its cognate RR CpxR and response of the cells. The Cpx system is inhibited by interaction of the periplasmic accessory protein CpxP with CpxA. As postulated by Kohanski et al. (2008) addition and uptake of aminoglycosides lead to mistranslation of proteins in the cytoplasm, and result in misfolded, periplasmic proteins activating the Cpx system [8]. According to this controversially discussed hypothesis, crosstalk between CpxA and ArcA takes place under this condition leading to cell death (red marked pathway). IM = inner membrane; OM = outer membrane

Kohanski et al. (2008) proposed within their study that treatment with 5 μg ml^−1^ gentamicin causes accumulation of misfolded proteins in the inner membrane and the periplasm resulting in hyper-activation of CpxA [8]. They concluded from their results that gentamicin treatment results in the accumulation of misfolded proteins in the inner membrane leading to radical formation and membrane depolarization. Kohanski et al. (2008) suggested that the Cpx-TCS is a key player in this process and that the SK CpxA cross-phosphorylates the RR ArcA [8]. Given that ArcA ~ P regulates several metabolic genes leading to free radical formation, Kohanski et al. (2008) concluded that this crosstalk between CpxA and ArcA finally results in cell death [8] (Fig. 1). In contrast to this, Mahoney & Silhavy (2013) showed that a dominant mutation of *cpxA* resulting in constitutive activation of the Cpx-TCS pathway in *E. coli* enhances the viability of cells grown in presence of 5 μg ml^−1^ gentamicin [3]. Increased viability was found to be CpxR-dependent although none of the tested Cpx regulon members was responsible for the monitored increased resistance against aminoglycoside antibiotics. However, increased viability in presence of aminoglycosides may be caused by the CpxR-dependent prevention from membrane damage induced by the misfolded proteins generated due to gentamicin treatment. In addition, it was found that CpxR is not required for cell death caused by bactericidal antibiotics [3]. Recently, it was demonstrated that cell death due to bactericidal antibiotics does not require oxygen or reactive oxygen species, thus further calling the hypothesis of Kohanski et al.’s (2008) model into question [45, 46].

To decipher these contrary findings, we aimed to investigate the postulated crosstalk between the Cpx- and the Arc-TCS at the molecular level in *E. coli* M1G655 wild type upon gentamicin treatment in comparison to bacteria grown in absence of gentamicin. Besides protein interaction experiments, relative quantification (gene expression) and absolute quantification (protein amounts) of the components of the Cpx- and the Arc-TCS were performed. For a deeper insight into alterations of the protein composition upon aminoglycoside treatment and the role of CpxA-dependent phosphorylation, global protein profiling experiments were performed on the *E. coli* MG1655 wild type and an isogenic *cpxAR* mutant.

## Results

### Cpx and ArcA interact specifically upon aminoglycoside treatment

**Fig. 2:**
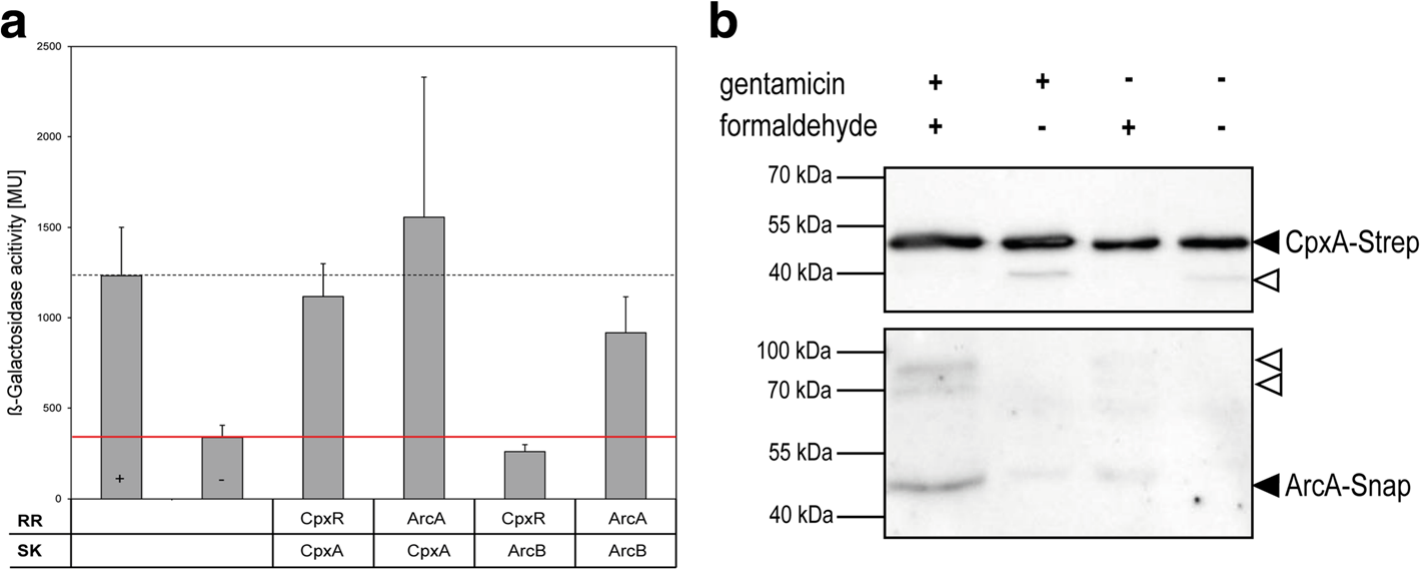
CpxA and ArcA interact in vivo. **a** Bacterial two-hybrid assay (BACTH) was used to identify interactions between CpxA and ArcA. The sensor kinases CpxA and ArcB were C-terminally fused to the T18-fragment, whereas the response regulators CpxR and ArcA were N-terminally fused to the T25-fragment of the *Bordetella pertussis* adenylate cyclase. Different combinations of sensor kinase and response regulator were co-overexpressed and the ß-galactosidase activity in Miller units [MU] was determined. CpxA/CpxR- and ArcB/ArcA-interactions serve as controls. Plasmids expressing fusions of T18 and T25 with leucine zipper domains of the transcription factor GCN4 derived from *Saccharomyces cerevisiae* (+) serve as a positive control. Empty vectors non-expressing the T18- and T25-fragment (−) serve as a negative control. The average of three biological replicates is shown. **b** The chromosomally-encoded fusion protein ArcA-Snap and plasmid-encoded CpxA-Strep were used to analyze interactions between CpxA and ArcA using Membrane-SPINE. Interactions were determined for cells treated with or without of 5 μg ml^−1^ gentamicin for 40 min. Additionally, samples without addition of the crosslinker formaldehyde or gentamicin served as controls. Out of two biological replicates, one representative blot is shown. Black triangles show specific bands of CpxA-Strep and ArcA-Snap, whereas white triangles represent unspecific bands

In order to investigate the postulated crosstalk between the Cpx- and Arc-TCS (CpxA with ArcA or ArcB with CpxR) after aminoglycoside treatment in *E. coli* ([8]; Fig. 1), we first analyzed the physical interactions between the sensor kinases (SK) CpxA and ArcB with the response regulators (RR) CpxR and ArcA of *E. coli* strain MG1655 in vivo. For this purpose, bacterial two-hybrid (BACTH) and Membrane-Strep-tagged protein interaction experiments (mSPINE) were performed **(**Fig. 2a,b). BACTH makes use of fusions of two putatively interacting proteins with two complementary fragments of *Bordetella pertussis* adenylate cyclase, namely T18 and T25. Interactions of proteins fused to T18 and T25 restore the abolished function of the adenylate cyclase, allowing detection and quantification of protein interactions by ß-galactosidase activity assay [47]. In this study, the SKs CpxA and ArcB were C-terminally fused to T18, whereas the RRs CpxR and ArcA were N-terminally fused to T25.

We co-overexpressed different combinations of SK and RR in an adenylate-cyclase-deficient strain and measured the ß-galactosidase activity for three biological replicates each (Fig. 2a). The ß-galactosidase activities for the natural SK/RR pairs CpxA/CpxR and ArcB/ArcA amounted to about 1100 Miller units (MU) and 800 MU, respectively (Fig. 2a). Interactions between CpxA and its non-cognate response regulator ArcA resulted in higher ß-galactosidase activity (about 1600 MU) than observed for the positive control (+) using fusions of T18 and T25 with leucine zipper domains of the transcription factor GCN4 derived from *Saccharomyces cerevisiae* (about 1200 MU; Fig. 2a). In contrast, interactions between ArcB and CpxR yielded lowest ß-galactosidase activity (about 250 MU) below the estimated background using empty vectors (about 400 MU) and thus no interaction could be identified for this SK/RR-pair (Fig. 2a). In sum, BACTH strongly suggested that physical interaction between CpxA and ArcA after overexpression of both interaction partners is possible without the need of any specific stimulus.

Next, we investigated whether CpxA and ArcA interact specifically due to aminoglycoside-caused stress. We therefore applied the Membrane-SPINE method under conditions almost identical to the experiments performed by Kohanski et al. (2008) [8]. Kohanski et al. (2008) used 5 μg ml^−1^ of the aminoglycoside gentamicin for aminoglycoside treatment and incubated the cells for 30 min [8]. In our study, cells were incubated for 40 min. The cross linker formaldehyde requires at least 20 min incubation time for reliable interaction data (Fig. 2b). The Membrane-SPINE approach combines the purification of a specific Strep-tagged membrane protein with the reversible fixation of protein complexes by adding the aforementioned cross linker formaldehyde, allowing snapshots of interactions in living cells [48]. Here we used a MG1655-derivate *E. coli* strain harboring a chromosomal fusion of the *arcA* gene with a Snap-Tag and a fusion of *cpxA* with a Strep-Tag on the medium copy plasmid pMal-p2X (~20 copies per cell; [49]). It was demonstrated in a previous study that CpxA-Strep is correctly localized in the inner membrane and fully functional for transphosphorylation of purified CpxR [49].

The interaction between CpxA-Strep and ArcA-Snap could be specifically observed after treatment with gentamicin and after addition of formaldehyde (Fig. 2b). Moreover, in samples treated with gentamicin but without formaldehyde or vice versa, only weak bands of ArcA-Snap were observed (Fig. 2b). The quantification of band densities revealed the highest band intensity for ArcA-Snap for samples treated with gentamicin and formaldehyde (Fig. 2b)**.** The amount of CpxA-Strep remained constant in all four samples (Additional file 1: Figure S1).

We proved that CpxA and ArcA interact physically and specifically after aminoglycoside treatment of the cells, fulfilling the first requirement for a crosstalk between the Cpx- and the Arc-TCS.

### Gentamicin treatment alters the expression levels of arcB and arcA to different extent

After verifying physical interaction of CpxA and ArcA in vivo, we hypothesized that aminoglycoside treatment could lead to a shift in ratios between CpxA, ArcB, and ArcA, thereby enhancing the probability of a crosstalk between CpxA and ArcA under physiological conditions. Kohanski et al. (2008) postulated Cpx activation after gentamicin treatment (Fig. 1 **,** [8]) and it is known that activation of the Cpx-TCS leads to increased amounts of CpxA [34]. Hence, crosstalk between CpxA and ArcA might be forced by a significantly increased amount of CpxA compared to that of ArcB. Moreover, the experiments by Kohanski et al. (2008) [8] as well as the experiments within this study were performed under aerobe, and thus Arc non-inducing conditions [39–44]. Therefore, an additional, significant deficiency of ArcB compared to CpxA could make crosstalk between CpxA and ArcA more likely. To address these aspects as well as to analyze the hypothesis of aminoglycoside driven Cpx activation, we estimated the expression levels of *cpxA*, *arcB*, and *arcA* first. For this purpose, we used identical growth conditions as described by Kohanski et al. (2008) [8].

**Fig. 3:**
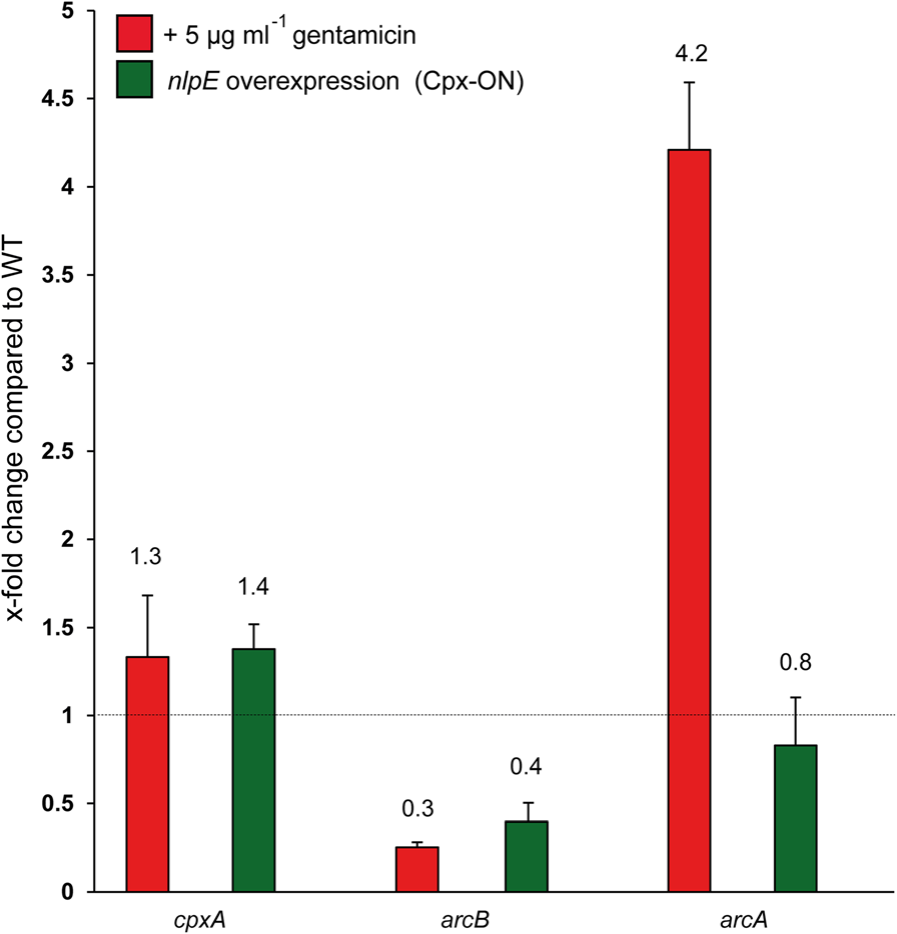
Impact of different stressors on the expression level of *cpxA*, *arcB,* and *arcA*. Changes in expression levels for the genes *cpxA*, *arcB,* and *arcA* were determined by q-RT-PCR after addition of 5 μg ml^−1^ gentamicin (red bars) and after *nlpE* overexpression (Cpx-ON; green bars). All depicted values represent ratios between the condition of interest and WT cells. Shown are mean data and standard deviations of five biological replicates measured in three technical replicates each

We performed quantitative reverse transcription-PCR (qRT-PCR) for *cpxA*, *arcB*, and *arcA* in *E. coli* MG1655 WT cells 30 min after addition of 5 μg ml^−1^ gentamicin and compared the expression levels with those from cells (i) grown under WT conditions (LB-medium), and (ii) upon activation of the Cpx system (overexpression of the outer membrane protein *nlpE*; Cpx-ON). The *nlpE* gene encodes the outer membrane lipoprotein NlpE and the overexpression of *nlpE* represents a well-known, Cpx-activating condition [34, 38]. The expression of *cpxA* increased (1.4-fold) after Cpx activation (Fig. 3). The expression of *arcB* and *arcA* behaved in opposite manners by decreasing after Cpx activation (Fig. 3). The expression of *arcB* decreased significantly after addition of gentamicin (0.3-fold), whereas the expression of *arcA* significantly increased in comparison to WT conditions (4.2-fold; Fig. 3). The significant changes in *arcB* expression match our initial hypothesis suggesting a deficiency of ArcB compared to CpxA after gentamicin treatment. However, the expression of *cpxA* changed only slightly after aminoglycoside treatment (1.3-fold). In awareness of the fact that expression levels of genes are not directly linked with activation and do not display the absolute amounts of proteins within the cell, we went one step further and quantified the absolute numbers of the Cpx- and the Arc-TCS proteins under different conditions.

### Absolute quantification of the Arc- and the Cpx-TCS

To further investigate if a possible crosstalk between the Arc- and the Cpx-TCS is indicated by altered amounts of respective molecules, we monitored the absolute number of proteins per cell upon gentamicin treatment. As controls, we also quantified the Arc- and the Cpx-TCS under WT conditions (LB-medium) and during Cpx-activating conditions (Cpx-ON by *nlpE* overexpression).

It is important to consider that protein translation is not fully blocked within 30 min of growth in presence of 5 μg ml^−1^ gentamicin as performed for the experiments in this study. This is reasoned in previous data demonstrating that cells still grow for at least 60 min in presence of sub-lethal gentamicin concentrations of 5 μg ml^−1^ [8]. Accordingly, the employed cells continued growing after addition of 5 μg ml^−1^ gentamicin in the present study (Additional file 1: Figure S2). Moreover, ribosome binding by aminoglycosides as gentamicin does not elicit immediate hold-up of translation, but mistranslation of proteins by false-incorporation of amino acids into the peptide strands [50].

We employed the single reaction monitoring (SRM) method which combines highly sensitive detection of selected molecules with exact and absolute quantification by spiking in heavy isotope labeled standard peptides of targeted proteotypic peptides from a desired protein [51, 52]. Recently, the SRM method was used to establish a global approach to absolutely quantify the *E. coli* proteome under different conditions [53]. These data also include absolute amounts of the Cpx- and the Arc-TCS. However, gentamicin treatment and Cpx activating conditions were not tested. The data for the present study are provided as Table 1, in which values labeled with * were recently published [34]. Even though differences were expected and existent between our study and Schmidt et al. (2016), the ranges of the determined copy numbers were comparable for bacteria grown in LB-medium [Schmidt et al. (2016); e.g. CpxA 41 molecules/cell our study, 50 molecules/cell Schmidt et al. (2016); ArcA 1088 molecules/cell our study, 5464 molecules/cell Schmidt et al. (2016)] [53].

**Table 1:** Absolute quantification of CpxAR, CpxP, and ArcBA by selected reaction monitoring

Peptide	Protein	Replicate	Average amount [molecules/cell]	SD [molecules/cell]	CV [%]
AEDSPLGGLR	CpxA	wild type conditions	41*	11	28
		with 5 μg ml^−1^ gentamicin	32	6	19
		*nlpE* overexpression (Cpx-ON)	78*	4	5
EHLSQEVLGK	CpxR	wild type conditions	393*	93	24
		with 5 μg ml^−1^ gentamicin	348	43	12
		*nlpE* overexpression (Cpx-ON)	738*	69	9
DVTQWQK	CpxP	wild type conditions	36*	15	42
		with 5 μg ml^−1^ gentamicin	41	17	42
		*nlpE* overexpression (Cpx-ON)	133*	28	21
FTQQGQVTVR	ArcB	wild type conditions	85	20	24
		with 5 μg ml^−1^ gentamicin	66	11	16
		*nlpE* overexpression (Cpx-ON)	82	8	10
SLIGPDGEQYK	ArcA	wild type conditions	1088	162	15
		with 5 μg ml^−1^ gentamicin	796	150	19
		*nlpE* overexpression (Cpx-ON)	484	41	8

The peptides used for absolute determinations of protein molecules per cell are given as average values from five biological replicates with their standard deviations and coefficient of variance (CV) in percent for each of the four applied conditions

Molecular amounts labeled with * represent data that were already published previously [32] but are mentioned here to provide complete information

**Fig. 4:**
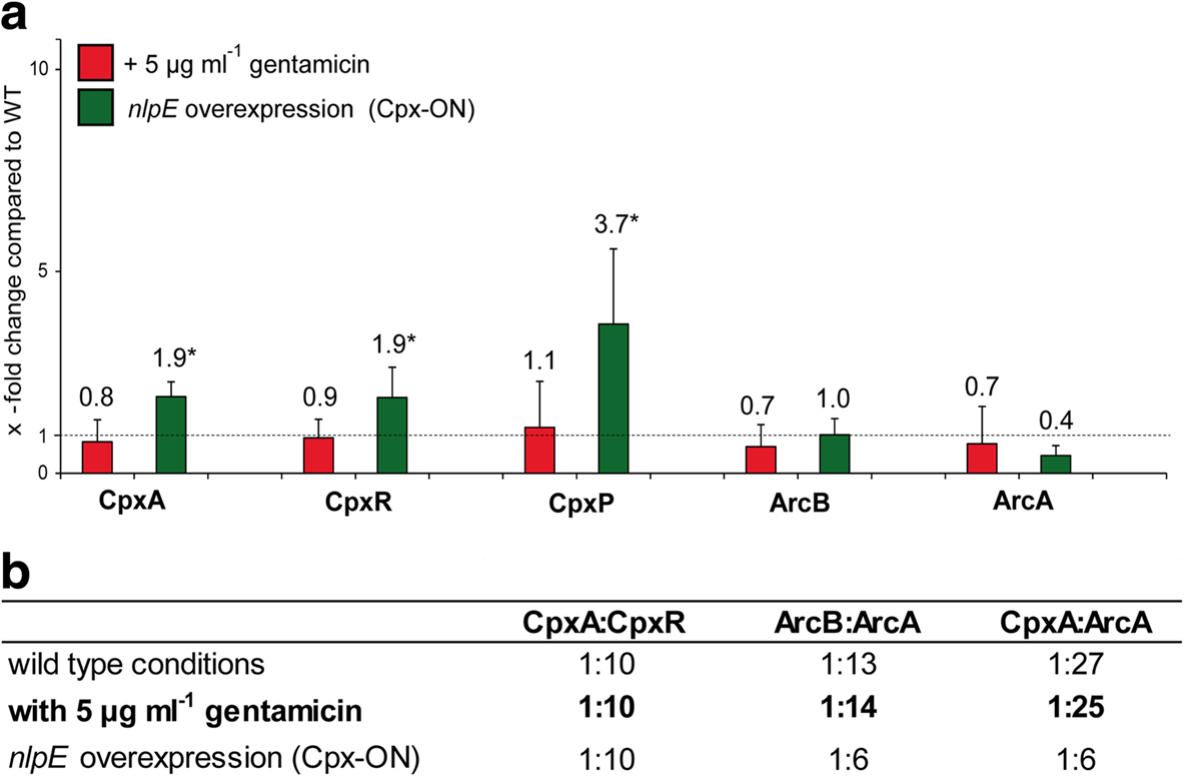
Absolute quantification of CpxAR, CpxP, and ArcBA under various conditions. A) Selected reaction monitoring (SRM) was used to determine the absolute amounts of CpxA, CpxR, CpxP, ArcB, and ArcA after addition of 5 μg ml^−1^ gentamicin (red bars) and after *nlpE* overexpression (Cpx-ON; green bars). All depicted values represent ratios between the condition of interest and WT cells. Shown are mean data and standard deviations of five biological replicates measured. Values labeled with * represent molecular amounts published in a previous study [34] but are listed here for complete information. B) The absolute amounts of CpxA, CpxR, ArcA, and ArcB were used to calculate the SK:RR-ratio for each tested condition

We provide values for ArcB and ArcA as well as for CpxA, CpxR, and the accessory protein CpxP under each tested condition. Similar to the qRT-PCR data, we observed changes in absolute protein amounts of the Cpx and Arc-TCS components upon Cpx activation and gentamicin treatment in comparison to native conditions (Fig. 4a). The absolute amounts of CpxA, ArcB and ArcA decreased after gentamicin treatment (Fig. 4a), disproving the initially raised hypothesis of a significant increase of CpxA and/or a significant decrease of ArcB supporting crosstalk between CpxA and ArcA.

Regarding cells grown under WT conditions, we observed a stoichiometry of SK to RR being CpxA:CpxR = 1:10, ArcB:ArcA = 1:13, and CpxA:ArcA = 1:27 (Fig. 4b **).** The determined ratios after addition of gentamicin were nearly the same (Fig. 4b), demonstrating a high robustness for both systems with regard to the stoichiometry. In contrast, activation of the Cpx-TCS (*nlpE* overexpression) significantly changed the ratio between CpxA and ArcA to 1:6 in comparison to 1:27 under native conditions.

Overall, the interaction between CpxA and ArcA distinctly observed in the Membrane-SPINE experiment was not caused by a shift in ratios between CpxA, ArcB, and ArcA in vivo.

### Alterations in the proteome profile of *E. coli* MG1655 upon gentamicin treatment

On the one hand, our results demonstrated gentamicin-dependent interaction between CpxA and ArcA. On the other hand, quantification of absolute protein amounts did not explain why these two proteins interact specifically upon gentamicin treatment. Kohanski et al. (2008) postulated a functional interaction between CpxA and ArcA, and Cpx activation upon gentamicin treatment on the basis of expression profiles of selected ArcA ~ P-dependent genes [8]. Here, we wanted to add information on the impact of the Cpx-TCS upon gentamicin treatment. Thus, we monitored the proteome profiles of *E. coli* MG1655 WT and an isogenic *cpxAR* mutant under control conditions (LB-media only) and upon gentamicin treatment for 30 min in four biological replicates (Additional file 2: Table S1). Avoiding the possibility of CpxA-mediated ArcA-phosphorylation in the absence of CpxAR allowed us to investigate if ArcA ~ P targets are differently affected in comparison to WT cells, pointing to Cpx-specific Arc-activation in the WT. Moreover, implementing the *cpxAR* mutant provides information about changes in protein levels being dependent on the presence of CpxAR and corresponding alterations in transcription, thereby rejecting background effects that are only caused by gentamicin treatment. Finally, phosphorylation of ArcA by ArcB can be neglected under the analyzed conditions as the Arc-TCS is commonly inactive under aerobic growth conditions [39–44].

Applying shotgun proteomics, we detected and quantified in total 1467 proteins in the dataset of the WT strain and the respective *cpxAR* mutant treated with or without gentamicin. Almost no alterations in ArcA or ArcB amounts were visible when comparing WT conditions and gentamicin treatment in both strains. In contrast, the Cpx-TCS seems to play an important role in the cell response upon gentamicin treatment.

**Fig. 5:**
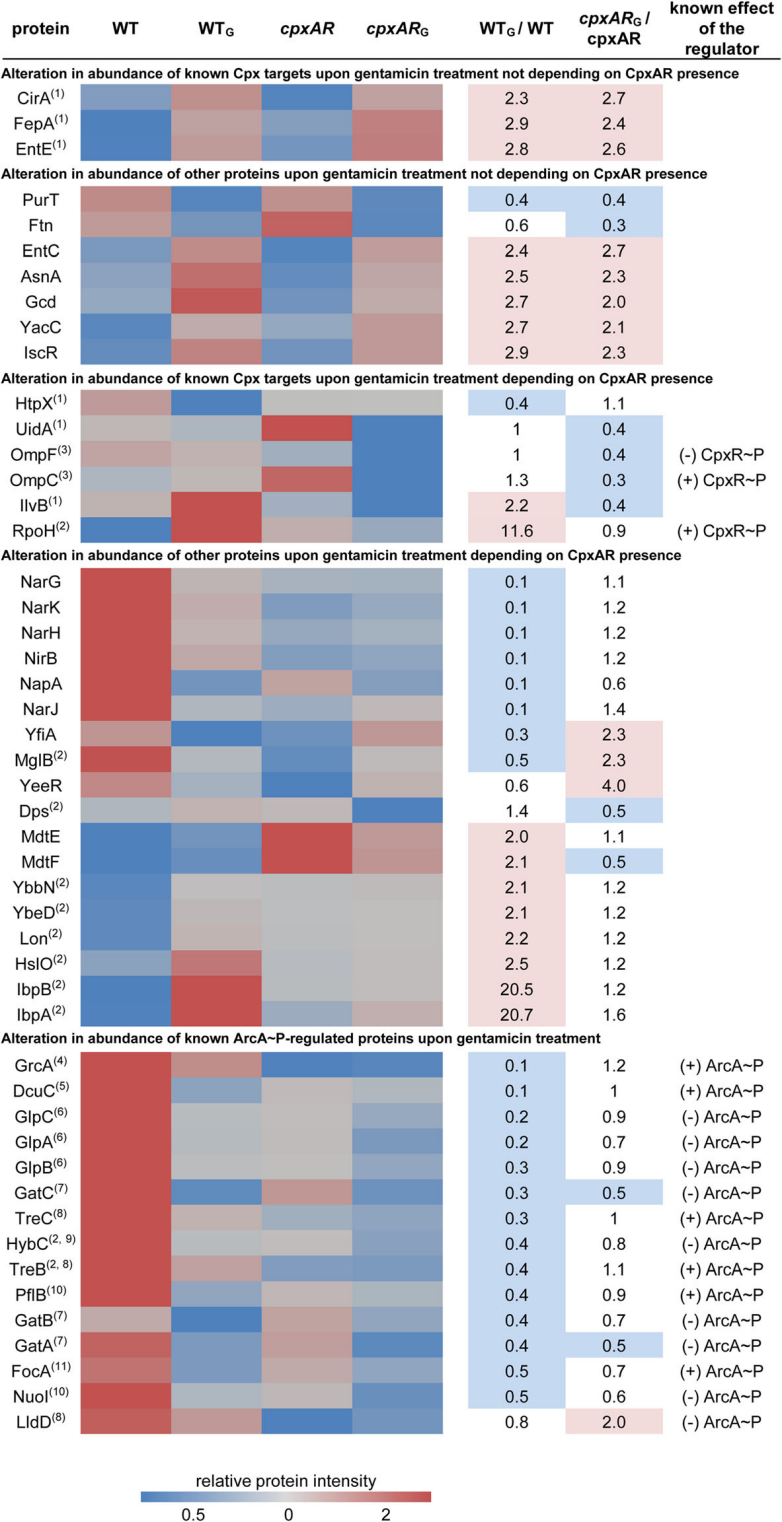
Altered protein abundance upon gentamicin (G) treatment in *E. coli* WT or *cpxAR* mutant cells. In the heatmap, relative proteins intensities are visualized, thereby red fields indicate higher relative intensities, dark blue lower relative intensities and grey fields show relative intensities around one. In addition, gentamicin treatment depending ratios between the WT and the *cpxAR* mutant are presented as well, ratios to the control >2 are labeled red, those <0.5 in blue. Proteins are grouped by CpxAR or ArcA dependence. Some proteins were already described to be Cpx targets [(1) Surmann et al. (2016) [34]], found regulated upon gentamicin on transcript level [(2), Kohanski et al. (2008) [8]], and/or positively (+) or negatively (−) CpxR ~ P or ArcA ~ P-regulated [(3) Batchelor et al. (2005) [69], (4) Wyborn et al. (2002) [70], (5) Zientz et al. (1999) [71], (6) Iuchi et al. (1990) [20], (7) Salmon et al. (2005) [72], (8) Liu & de Wulf (2004) [44], (9) Richard et al. (1999) [73], (10) Shalel-Levanon et al. (2005) [74], (11) Kaiser & Sawers (1997) [75]]

In WT cells, 115 proteins were significantly altered in their level after gentamicin treatment compared to non-treated WT. In the *cpxAR* mutant this was only true for 39 proteins when comparing gentamicin to control conditions (Additional file 2: Table S1). A selection of proteins already affected by aminoglycoside addition after 30 min is provided in the heatmap in Fig. 5 visualizing relative protein intensities in all tested conditions as well as the impact of gentamicin on the WT (WT_G_/WT) or the *cpxAR* mutant (*cpxAR*_G_/*cpxAR*).

An initiated inhibition of protein translation by gentamicin is partly visualized by lower intensities of some proteins upon gentamicin treatment (Fig. 5). Nevertheless, a CpxAR-dependent adaptation to gentamicin could be observed. Only three of these proteins with different behavior in the WT and the mutant strain contain binding sites for CpxR ~ P at the DNA level. These were the two outer membrane proteins OmpF and OmpC with decreased levels in the mutant and slight alteration in the WT upon gentamicin treatment. The third protein with CpxR ~ P binding site at the DNA level, the RNA polymerase heat shock sigma factor RpoH, was strongly increased in WT cells (WT_G_/WT 11.6) and not changed in level regarding the mutant. A CpxAR-dependent increase in protein amounts upon gentamicin treatment was further shown for the two heat shock proteins IbpA and IbpB to high extent (WT_G_/WT 20.7 and 20.5), and to a lesser extent for the two multidrug efflux pump proteins MdtE and MdtF (WT_G_/WT 2.0 and 2.1), as well as for the Lon protease (WT_G_/WT 2.2). Finally, proteins of the Nar family (respiratory nitrate reductases) were found to be decreased upon gentamicin treatment (WT_G_/WT < 0.2). Hence, we found remarkable changes in the global proteome profiles comparing the WT- and the *cpxAR* strain (single comparison WT_G_/WT vs. *cpxAR*_G_/*cpxAR*).

Some proteins were altered in level independently of CpxAR presence after gentamicin treatment as indicated by similar ratios of WT_G_/WT compared to *cpxAR*_G_/*cpxAR.* These displayed changes caused by gentamicin without any measurable Cpx-involvement. Importantly, monitoring not only decreased protein levels due to initiated translation inhibition triggered by gentamicin, but also increased protein levels indicate intact transcription and translation within the observed growth period and gentamicin concentration. Among these were also three proteins which were already identified as Cpx targets [34]: the enterobactin synthetase component E (EntE) and two iron-dependent transport proteins, the ferric iron-catecholate outer membrane transporter (CirA) and the ferrienterobactin receptor (FepA). These proteins were likewise increased in WT and *cpxAR cells* upon gentamicin treatment. Other Cpx-independent increased protein levels were found e.g. for the transcription regulator IscR and some metabolic enzymes such as the aspartate-ammonia ligase AsnA, the glucose dehydrogenase (Gcd), and the isochorismate synthase EntC as well as the uncharacterized protein YacC. Lower levels upon aminoglycoside uptake in both strains were found for the galactitol-specific enzyme IIA component of PTS (GatA) and the transferase PurT.

Twelve of 15 detected ArcA ~ P-regulated proteins were found significantly decreased in level upon gentamicin treatment in the WT strain. In contrast, gentamicin had no effect on most ArcA ~ P targets in the *cpxAR* mutant (Fig. 5). Among the ArcA ~ P targets identified in the WT strain were for example three anaerobic glycerol-3-phosphate dehydrogenases GlpA, GlpB, and GlpC, the anaerobic C4-dicarboxylate transporter DcuC, and two trehalose metabolizing enzymes TreB and TreC. Only GatC and GatA were likewise decreased in the mutant, and LldD was doubled in amount in the strain lacking *cpxAR*. Although a Cpx-dependent effect on the ArcA ~ P targets can be clearly seen, this is most likely not a result of CpxA-mediated ArcA phosphorylation, as these targets were not altered according to known regulation (positive or negative) via ArcA ~ P.

Our results indicated that CpxA does not cross-phosphorylate ArcA. To investigate this further, we determined the ArcA isoform pattern by 2D gel electrophoresis for WT conditions, after Cpx activation (*nlpE* overexpression, Cpx-ON) and upon gentamicin addition. We did not observe any ArcA phosphorylation in these samples. Unfortunately, aspartate phosphorylations as described for ArcA ~ P [54] are known to be very unstable. Using 2DE gels we were able to detect ArcA as the prevailing protein in two spots under all conditions at the predicted mass range and separated from each other by a pH shift into the acid milieu pointing to the expected phosphorylation, which, however, could not be confirmed by standard nanoLC-MS/MS. Since the spot volume was comparable under WT conditions and upon gentamicin treatment (example 2D gel shown as Additional file 1: Figure S3) no hints for an alteration of the ArcA status could be found.

### The proteome profile after classical Cpx activation differs significantly from the proteome composition after gentamicin treatment

To elucidate if gentamicin induces the Cpx system as hypothesized by Kohanski et al. (2008) [8] and demonstrated by Kashyap et al. (2011) on transcriptomic level [55], we compared proteome profiles of cells treated with gentamicin with a previously published proteome profile of cells grown under Cpx activating conditions via overexpression of *nlpE* [34]. Therefore, the proteome data acquired within this study was re-evaluated essentially as performed for the proteome profiles acquired after Cpx activation by *nlpE* overexpression [34]. Here, employment of the (WT_G_/WT)/(*cpxAR*_G_/WT)-ratio emphasizing higher amounts of respective proteins WT_G_ cells compared to *cpxAR*_*G*_ cells revealed only eleven proteins as Cpx-dependently increased after gentamicin treatment. Among these were proteins involved in metabolism: FruK [(WT_G_/WT)/(*cpxAR*_G_/WT): 16.7], FruA (13.8), IlvB (8.1), or stress response: IbpB (10.1), IbpA (9.7), RpoH (5.3), YgiQ (5.1), YceA (4.1), YncD (3.1), YrbL (2.2), and DeaD (2.0) (Additional file 3: Table S2). In contrast, we found seven proteins with decreased levels after addition of gentamicin depicted by the (*cpxAR*_G_/WT)/ (WT_G_/WT)-ratio that underlines higher amounts of the respective protein in absence of CpxAR after gentamicin treatment. These proteins were YodD [(*cpxAR*_G_/WT)/(WT_G_/WT): 8.7], GarR (3.2), YfiA (3.0), YoaC (2.9), PptA (2.5), GcvP (2.2), and PhnA (2.0). None of the depicted proteins strongly suggesteds a binding site for ArcA ~ P or CpxR ~ P, except for RpoH harboring a binding site for CpxR ~ P, as asserted previously. Furthermore, the protein IlvB is the only protein that was identified in a previous study being specifically increased after Cpx activation by *nlpE* overexpression [34]. Hence, missing or low Cpx activation could be verified by comparing these two data sets.

Further comparison of the two protein profiles (gentamicin addition versus *nlpE* overexpression) revealed that NlpE-mediated Cpx activation caused increase of the proteins AceA, GltA, and FadB, and decrease of the proteins GadA, CydA, and CydB [34]. All of these proteins are known to be regulated by ArcA ~ P at the transcriptional level, but in an opposite manner to Cpx, thereby being negatively regulated by ArcA ~ P as it holds true for e.g. *aceA*, *gltA*, and *fadB*. Hence, Cpx activation itself seems not to pronounce putative crosstalk to ArcA, since the Cpx response dominated in regulation of the aforementioned proteins.

### Protein abundance correlation studies of the Cpx- and the arc-TCS revealed significant correlations to various chaperone proteins

Finally, we wanted to identify proteins whose protein levels correlate to the protein levels of CpxA and ArcA in order to investigate to what extent CpxA and ArcA affect the protein pattern of *E. coli* MG1655 WT cells. Therefore, we profiled the proteome under control conditions (WT), Cpx activation by *nlpE* overexpression (Cpx_ON_) as well as after treatment with gentamicin (WT_G_) and analyzed the correlation of protein abundances of all 1462 detected proteins with CpxA and ArcA amounts applying a linear model. Proteins whose levels increased likewise to those of CpxA or ArcA showed a high correlation with the maximum value of 1. In contrast, proteins whose levels decreased to the same extent as the target proteins increased obtained negative values (defined as significant for correlation values >0.8 or < −0.8). The results revealed a significant negative correlation between ArcA and CpxA/CpxR and a significant positive correlation between CpxA- and CpxR-levels (Additional file 4: Table S3). No significant correlation between the amounts of RR ArcA and its SK ArcB was detected, indicating that the ArcB-ArcA interaction might not be relevant under the applied aerobic conditions. Further, amounts of at least 15 annotated proteins with chaperone function were found to be significantly associated with the amounts of ArcA, ArcB, CpxA or CpxR. Four chaperons were significantly correlated with ArcA and CpxAR (Additional file 4: Table S3). In contrast to the proteins of the Cpx-TCS, these chaperones (Skp, SurA, GroL, and ClpA) showed specifically regulated protein levels during Cpx activation by *nlpE* overexpression, gentamicin treatment and WT conditions. The levels of Cpx- and Arc-TCS proteins did not change between gentamicin and WT conditions (Additional file 4: Table S3).

## Discussion

The effect of the Cpx-TCS upon gentamicin treatment has been controversially discussed. Kohanski et al. (2008) postulated a gentamicin-induced crosstalk between the SK CpxA and the RR ArcA resulting in cell death [8]. This hypothesis was based on monitoring CpxA-dependent expression of ArcA ~ P targeted genes independently of CpxR. In contrast, Mahoney and Silhavy (2013) showed that the Cpx system protects cells from aminoglycoside antibiotics and hydroxyurea [3]. Mahoney and Silhavy (2013) were able to demonstrate that survival in presence of 5 μg ml^−1^ gentamicin strictly requires CpxR [3]. However, they were not able to identify the respective Cpx regulon member targeted by CpxR in presence of gentamicin.

We demonstrated in our study that physical interaction between CpxA and ArcA is possible without any kind of stimulus. This occurs under conditions overexpressing both interaction partners, as it was the case for the performed BACTH interaction analyses. However, expressing one interaction partner from the chromosome (ArcA-Snap) in combination with a mid-copy plasmid-derived CpxA-Strep variant using the mSPINE approach, we were even able to demonstrate that the interaction is dependent on the presence of 5 μg ml^−1^ gentamicin. One theory raised within this study was that a significant increase of CpxA and/or a significant decrease of ArcB in presence of gentamicin might promote interaction between CpxA and ArcA instead of the cognate ArcB-ArcA interaction. A significant decrease of *arcB* expression accompanied by constant expression levels of *cpxA* after gentamicin treatment supported this theory at least partially. However, with respect to the absolute quantification of the Cpx- and Arc-TCS on the protein level after gentamicin treatment, this hypothesis was rejected as the absolute amounts of CpxA, ArcB and ArcA decreased. These differences between the transcriptional and the protein level emphasize the need to combine expression and protein data to fully consider post-transcriptional and post-translational effects. Nevertheless, it is important to note that the CpxA:ArcA-ratios displayed 1:6 after Cpx-TCS activation by *nlpE* overexpression switching to 1:25 after gentamicin treatment. Since the CpxA:CpxR-ratio remained constant among all conditions being 1:10, RR-competition between CpxR and ArcA is higher upon gentamicin presence. It remains unclear whether these changes alone enable CpxA-ArcA interaction upon gentamicin treatment.

In the second part of our analyses, we focused on the output of the CpxA-ArcA interaction upon gentamicin treatment. Based on the fact that Kohanski et al. (2008) found a CpxA-dependent expression of ArcA ~ P targeted genes [8], we aimed to decipher such effects on the protein level. Furthermore, we addressed the question whether gentamicin treatment provokes Cpx activation.

Gentamicin treatment caused significant Cpx-dependent changes in the global proteome profile of *E. coli* MG1655. For a dozen proteins being regulated by ArcA ~ P at the DNA level, we could detect a Cpx- and Gentamicin specific alteration. However, this alteration did not correlate to the kind of ArcA ~ P regulation being positive, or negative, respectively. This finding was supported by the fact that ArcA is most likely not phosphorylated after gentamicin treatment as visualized by 2D gel electrophoresis.

Nevertheless, we could observe that the Cpx system is highly involved in modulating the global proteome profile in presence of gentamicin. This became evident in several proteins being significantly altered in a Cpx-dependent manner. These included the strongly increased protein RpoH. Its relation to the activation of CpxR during stress in *E. coli* was shown previously [56]. It has already been demonstrated that accumulation of unfolded proteins induces the production of heat shock proteins as RpoH [57]. Further, it has been discussed that RpoH may be additionally stabilized under this condition by a passive mechanism: The chaperones DnaK, DnaJ, and GrpE could be titrated away from RpoH in presence of unfolded proteins instead of interacting with RpoH promoting its degradation [58]. Nevertheless, observing changes in levels of some proteins as RpoH only in presence of CpxAR proves additional involvement of the Cpx-TCS by an unknown mechanism. Interestingly, two multidrug efflux pump proteins were also increased Cpx-dependently after gentamicin treatment. Multidrug efflux pumps contribute to the resistance of *E. coli* towards antibiotics via export of the respective substances [59]. Moreover, MdtE, MdtF and the outer membrane channel TolC are known to promote increased tolerance to different ß-lactam antibiotics [60]. Previous transcriptomic data identified the operons encoding the NADH dehydrogenase Nuo, the ferrous iron transporter EfeUOB, the succinate dehydrogenase SQR, and the cytochrome *bo* terminal oxidase complex as downregulated by CpxR ~ P [33]. Furthermore, knock-out mutants of the first gene corresponding to each of the named operons increased the cell viability in the presence of 3 μg ml^−1^ of the aminoglycoside amikacin [33]. Within the data presented here, proteins referring to these operons were not significantly altered after gentamicin treatment, except for NuoI (WT_G_/WT 0.5). This might be due to employing another aminoglycoside in a different concentration and in a different *E. coli* strain background. However, monitoring the Cpx-dependent increase of MdtE and MdeF after gentamicin treatment might represent a protective mechanism against aminoglycoside antibiotics controlled by the Cpx-TCS. This is in contrast to the postulated Cpx- and Arc-mediated cell death under this condition [8], but in line with Mahoney & Silhavy (2013) proposing a protective role for the Cpx system in presence of aminoglycosides [3].

Moreover, our results strongly suggest that the Cpx system is not activated in presence of gentamicin. We found that the absolute amounts of CpxA, CpxR, and CpxP changed to a lower extent upon gentamicin addition compared to alterations upon well-described Cpx activating conditions (*nlpE* overexpression). Regarding the global proteome, only three proteins with altered protein abundance in dependency of CpxAR presence possess a binding site for CpxR ~ P at the DNA level. Cpx activation after gentamicin treatment became even more implausible after comparing the proteome profiles acquired after gentamicin treatment and *nlpE* overexpression which differed significantly. Here, the question whether and how these findings match previous data published by Kohanski et al. (2008) deserves further contemplation [8]. First, we can conclude that the putative interaction between CpxA and ArcA is most likely not dependent on Cpx activation, but most likely on gentamicin addition. This is reasonable considering that Cpx activation by *nlpE* overexpression evoked increased abundance of some proteins known to be negatively regulated by ArcA ~ P and vice versa. Second, response regulator competition is one well-known mechanism to prevent crosstalk [61]. Matching this mechanism, increase of CpxR with simultaneous decrease of ArcA upon *nlpE* overexpression implies that CpxR dominates regarding CpxA-mediated phosphorylation under this particular condition. This is of high importance taking into account that ArcA ~ P not only regulates some Cpx targets in an opposite manner, but also that a crosstalk between the Cpx- and the Arc-TCS is postulated to be lethal, and thus, should be avoided. Moreover, it is in general disputable how the Cpx system should be able to differ between activating conditions obviously avoiding crosstalk to the Arc-TCS (*nlpE* overexpression), and activating conditions enforcing crosstalk to the Arc-TCS (gentamicin treatment), further pointing to CpxA-ArcA interaction independently of the Cpx-active state. For future studies, it would be interesting to address whether activation of the Cpx system by e.g. *nlpE* overexpression promotes interactions between CpxA and ArcA.

Summarizing our overall results we suppose that the interaction between CpxA and ArcA monitored specifically after aminoglycoside treatment does not point to a cross-phosphorylation of ArcA via CpxA and subsequently to activation of the Arc system by CpxA. Nevertheless, our data support the hypothesis of Kohanski et al. (2008) [8], as an intersection of these two TCSs could be found by proteomic analyses. In contrast to Kohanski et al. (2008) [8], we identified protein levels of multidrug efflux pump proteins as Cpx-specifically increased in presence of gentamicin. This is more of an argument for protection against aminoglycosides than for Cpx- and Arc-mediated cell death, confirming previously recorded data [3, 33]. Altogether, our data point to an unknown, but distinct involvement of CpxAR upon gentamicin presence.

## Conclusions

Gentamicin caused pronounced changes in the global proteome profile of *E. coli*, which were found to be partially dependent on the presence of CpxAR. These alterations occurred despite the fact that the Cpx-TCS seems to be not, or to a lesser extent, activated by gentamicin treatment compared to classical Cpx activating conditions like *nlpE* overexpression. Nevertheless, CpxA and ArcA were found to specifically interact upon gentamicin addition. However, this interaction seems not to be relevant for CpxA-mediated phosphorylation and activation of ArcA, although an intersection of these two pathways could be found by proteomic analyses. While the type of intersection between these two pathways remains enigmatic, the results of the present study increase the understanding of a complex regulatory network in the *E. coli* response to aminoglycoside caused stress and on the complex network of TCSs in *E. coli*.

## Methods

### Bacterial strains and plasmids

All *E. coli* strains and plasmid used in this study are described in Table 2. Strains were grown in Luria-Bertani (LB) medium [62]. When necessary, antibiotics were included at the following concentration: 150 μg ml^−1^ampicillin, 5 μg ml^−1^ gentamicin.

**Table 2:** *E. coli* strains and plasmids used in this study

Strain / plasmid	Relevant genotype	Reference or source
MG1655	F-lambda- *ilvG*- *rfb*-50 *rph*-1	Blattner et al. (1997) [68]
EMC07E	F-lambda- *ilvG*- *rfb*-50 *rph*-1 *cpxAR::kan*	Surmann et al. (2016) [34]
GP01E	F-lambda- *ilvG*- *rfb*-50 *rph*-1, *arcA*-Snap	this work
BTH-101	F-, *cya-99*, *araD139, galE15, galK16, rpsL1,* *(Str r)*, *hsdR2, mcrA1, mcrB1*	Euromedex, Souffelweyersheim, France
pT*nlpE*	*nlpE* cloned in pTrc99A, Amp^R^	Zhou et al. (2011) [24]
pKT01E	CpxA-Strep on pMal-p2X without MalE, Amp^R^	Tschauner et al. (2014) [49]
pUT18	T18-fragment (amino acids 225–399 of *cyaA*), *lac* Promotor, MCS located at 5′-end of T18; in pUC19 (high copy number), Amp^R^	Euromedex
pKT25	T25-fragment (amino acids 1–224 of *cyaA*), *lac* Promotor, MCS located at 3′-end of T25; in pSU40 (low copy number), Kan^R^	Euromedex
pSH106E	*cpxA* in pUT18, Amp^R^	this work
pSH105E	*arcB* in pUT18, Amp^R^	this work
pEL16E	*cpxR* in pKT25, Kan^R^	this work
pGP02E	*arcA* in pKT25, Kan^R^	this work
pKT25-zip	leucine zipper of GCN4 from *Saccharomyces cerevisiae* in pKT25, Kan^R^	Euromedex
pUT18C-zip	leucine zipper of GCN4 from *Saccharomyces cerevisiae* in pUT18C, Amp^R^	Euromedex

### Harvest of bacterial culture

*E. coli* cells were diluted 1:100 from an overnight culture and grown aerobically at 37 °C in LB medium to an optical density at 600 nm (OD_600_) of ~0.5. Subsequently, IPTG (isopropyl-ß-D-thiogalactopyranoside) was added to a final concentration of 1 mmol l^−1^ to induce the overexpression of *nlpE* (pT*nlpE*) in MG1655. For crosstalk-inducing conditions, gentamicin was added in a final concentration of 5 μg ml^−1^. After additional growth for 30 min to an OD_600_ of ~1, cells were harvested by centrifugation at 5000 x g for 10 min and immediately frozen at −80 °C. The number of cells per milliliter cell culture was assigned using light microscopy and a Thoma chamber.

### Analysis of CpxA- and ArcA-interaction in vivo by bacterial two-hybrid (BACTH)

The method of bacterial two-hybrid was performed as described by Karimova et al. (1998) [47]. Bacterial two-hybrid (BACTH) is based on fusions of two putative interacting proteins with two complementary fragments of *Bordetella pertussis* adenylate cyclase, namely T18 and T25. T18 and T25 build up the catalytic domain of the adenylate cyclase CyaA which converts ATP to cAMP. Separation of T18 from T25 leads to the loss of adenylate cyclase function and transcriptional activation of e.g. catabolic operons mediated by cAMP together with CAP (catabolite activator protein). Interactions of proteins fused to T18 and T25 can restore the function of the adenylate cyclase in a strain lacking the *cya* gene. Cyclic AMP can be generated again and the transcription of several genes as the *lac*- or *mal*-operon can be activated. In this study, *cpxA* and *arcB* derived from *E. coli* were cloned into the vector pUT18 containing the T18-fragment resulting in C-terminal fusions of CpxA (pSH106E) and ArcA (pSH105E) with T18. The *cpxR*- and *arcA* genes were cloned into pKT25 containing the T25-fragment leading to N-terminal fusions of CpxR (pEL16E) and ArcA (pGP02E) with T25. These combinations of N- and C-terminal fusions were chosen as the N-terminal domain of a SK contains the input domain [63]. Furthermore, it is not known whether N-terminal fusions to e.g. CpxA allow for correct integration of this fusion protein into the inner membrane as this failed for a N-terminal CpxA fusion protein variant [64]. Contrarily, the C-terminal domains of RRs often contain the output domain essential for binding to respective promoter regions [65]. *E. coli* cells of the strain BTH-101 lacking the adenylate cyclase were co-transformed with different combinations of the described plasmids. After transformation, cells were grown aerobically in LB-Medium (pH 7) to an OD_600_ of ~0.6. Putative protein interactions were quantified by determination of the ß-galactosidase activity measuring the color shift of the substrate homologue *ortho*-nitrophenyl-ß-galactoside (ONPG) from colorless to yellow after cleavage.

### Analysis of CpxA- and ArcA-interaction in vivo by membrane-SPINE

Membrane-SPINE was performed as described previously with min minor modifications [48]. In brief, GP01E cells (chromosomal fusion of *arcA* with Snap-tag in MG1655) were transformed with pKT01E harboring a C-terminal fusion of *cpxA* with Strep-tag. Cells were grown in 520 ml LB (pH 7) supplemented with ampicillin at 37 °C until OD_600_ ~ 0.7.The expression of *cpxA*-Strep was induced by addition of IPTG (isopropyl-ß-D-thiogalactopyranoside) in a final concentration of 0.5 mmol l^−1^. After growth until OD_600_ ~ 0.9, cells were split and either non-treated or treated with gentamicin in a final concentration of 5 μg ml^−1^. After additional incubation for 20 min with or without gentamicin, formaldehyde was added in a final concentration of 0.6%, whereby samples without addition of formaldehyde served as an additional control. Cells were harvested after additional 20 min of incubation by centrifugation (3000 x g for 30 min at 4 °C) and resuspended in 16.6 ml buffer P1 (20 mmol l^−1^ Tris-HCl, 0.5 mol l^−1^ sucrose, pH 8.0). By addition of 2 ml buffer P2 (2 mg ml^−1^ lysozyme in 0.1 mol l^−1^EDTA, pH 7.5) spheroplasts were generated and afterwards collected by centrifugation (10,000 x g, 30 min, 4 °C). Spheroplasts were resuspended in 6 ml buffer P3 (20 mmol l^−1^ Tris-HCl, 0.1 mmol l^−1^ PMSF, pH 8.0) and subsequently disrupted by ultrasonication (Branson Sonifier 250, Emerson Industrial Automation, Ferguson, MO, USA) on ice (five pulses, each 30 s). Cell debris and unbroken cells were removed by centrifugation (10,000 x g, 10 min, 4 °C). After ultracentrifugation of the supernatant (100,000 x g, 30 min, 4 °C), the resulting pellet containing the membrane fraction [5 mg ml^−1^ membrane proteins; measured using the Implen P330 Nanophotometer (Implen, München, Germany)] was resuspended in buffer P3 supplemented with 2% dodecyl maltoside (DDM, Glycon, Luckenwalde, Germany) and stirred on ice for 1 h. Non-solubilized proteins and solubilized proteins were separated by ultracentrifugation (100,000 x g, 30 min, 4 °C). CpxA-Strep was purified using a Strep-tactin column (1 ml Superflow-StrepTactin sepharose, IBA, Göttingen, Germany). The column was equilibrated with buffer W (100 mmol l^−1^ Tris-HCl, 150 mmol l^−1^ NaCl, 1 mmol l^−1^ EDTA, 0.05% DDM, pH 8.0) and washed with 20 column volumes buffer W. Afterwards, CpxA-Strep and chemically crosslinked proteins were eluted with buffer E (100 mmol l^−1^ Tris-HCl, 150 mmol l^−1^ NaCl, 1 mmol l^−1^ EDTA, 2.5 mmol l^−1^ Desthiobiotin, 0.05% DDM, pH 8.0). Using a centrifugal filter unit (Amicon YM10 filter device, Milipore) elution fractions were 10-fold concentrated. The samples were boiled at 95 °C for 20 min to remove crosslinks. Proteins were separated by SDS-PAGE and analyzed by immunoblotting. CpxA-Strep and ArcA-Snap were detected using the primary antibody α-Strep-MAB-classic (Iba, Göttingen, Germany) in a working dilution of 1:10,000 and the antibody α-SnapTag-rabbit IgG (NEB, Frankfurt am Main, Germany) in a working dilution of 1:1000, respectively. Protein band visualization was carried out using SuperSignal West Pico chemoluminescent Substrate ECL-kit (Thermo Scientific Pierce Protein Biology Products) with a peroxidase-conjugated anti-rabbit IgG antibody (GE Healthcare) in a working dilution of 1:5000 and the ChemiDoc™ MP imaging system with Image Lab™ software (BIO-RAD, München, Germany).

### Quantitative reverse transcription-PCR (qRT-PCR)

The expression levels of the genes *cpxA*, *arcB,* and *arcA* were analyzed by quantitative reverse transcription-PCR (qRT-PCR) using five biological replicates for each condition. Every biological replicate was tested with three technical replicates. The extraction of total RNA was performed using the RNeasy minikit (Qiagen, Hilden, Germany) according to manufacturer’s instructions. After dilution of the RNA to a final concentration of 20 ng μl^−1^ and digestion of residual DNA contaminations by DNase I, cDNA was synthesized via the RevertAid First Strand cDNA synthesis kit (Fermentas). The qRT-PCR experiments were performed according to the following protocol: The first cycle (95 °C/2 min) was followed by forty repeats of Cycle 2 including several heating steps (95 °C/15 s; X °C/30 s; 72 °C/30 s). Temperature X displays the primer-specific annealing temperature. The protocol was completed by three more cycles: 95 °C/1 min, 55 °C/1 min, and 55 °C/10 s. The last cycle includes a temperature increment of 0.5 °C after every 10 s to enable melt curve data collection and real-time analysis. The primer pairs used in this study are CpxA-qRT_fw2/CpxA-qRT_rev2 annealing in *cpxA* (TCT GTT CCG GGC GAT TGA TA and TTA TCT TCG CCA TCA CGC AC), ArcB-qRT_fw/ArcB-qRT_rev annealing in *arcB* (ACT GGA GGA GTC ACG ACA AC and TGT GTC TCT TCG CGC TCT TT) and ArcA-qRT_fw/ArcA-qRT_rev annealing in *arcA* (GGC GAA TGT TGC GTT GAT GT and AGC TTT CAA CGC TAC GAC GT). Since an internal standard is needed we used the primer pair GapA-qRT-fw/GapA-qRT-rev (CTC CAC TCA CGG CCG TTT CG and CTT CGC ACC AGC GGT GAT GTG) amplifying the *E. coli* housekeeping gene *gapA* (glyceraldehyde-3-phosphate dehydrogenase A). The levels of expression of *cpxA*, *arcB,* and *arcA* were normalized to the expression level of *gapA* and the x-fold change of the expression after Cpx activation compared to WT cells was calculated.

### Preparation of protein extracts

As described before [34], bacterial cell pellets from 10 ml culture harvested at an OD_600_ of ~1 were reconstituted in 150 μl buffer containing 8 mol l^−1^ urea and 2 mol l^−1^ thiourea and disrupted by ultrasonication (50 W, 3 × 30 s, on ice, SonoPuls, Bandelin electronic, Berlin, Germany). After centrifugation (20,000 x g, 4 °C, 1 h) the protein concentration of the supernatant was determined using a Bradford assay (Biorad, Munich, Germany). Absolute amounts of protein per *E. coli* cell were determined by counting the number of bacteria per ml cell culture in a Thoma chamber using light microscopy and afterwards the total protein amount was correlated to the bacterial counts. Applying this method revealed the cellular amount of protein of *E. coli* K12 to be 1.4 × 10^−7^ μg (average from four conditions and five biological replicates).

### Heavy spike-in for absolute quantification by SRM

Two heavy labeled (^13^C and ^15^N arginine and lysine) proteotypic peptides for ArcA and three peptides for ArcB were obtained from JPT (JPT, Berlin, Germany). The peptide setup for the proteins of the Cpx-TCS was published recently [34]. In this work we refer to these data for the Cpx-TCS to compare the results to the newly acquired Arc-TCS data. The standard peptides possessed a C-terminal amino acid tag which was needed for quantification during manufacturing. It was eliminated by tryptic digestion during sample processing for mass spectrometry. The peptides were obtained as lyophilized powder and each one nmol was reconstituted in 100 μL buffer comprising 80% (*v*/v) aqueous ammonium bicarbonate solution (100 mmol l^−1^) and 20% (v/v) ACN. Prior to usage the peptide solutions were stored in aliquots of 10 μl at −80 °C. Pure peptide solutions were tryptically digested and analyzed by shotgun MS and SRM to control incorporation rate and purity of the peptides. All peptides were fully labeled and the share of contamination (trypsin and keratin) amounted to <1%. For method optimization and absolute quantification of the proteins, standard peptides were spiked into sample background and digestion occurred as described below.

### Protease digestion in solution

Four μg protein from each sample were diluted in 20 mmol l^−1^ aqueous ammonium bicarbonate solution to a final urea concentration below 2 mol l^−1^. Prior to SRM analyses, heavy peptides were added to the sample in this step. Proteins were reduced with 2.5 mmol l^−1^ dithiothreitol at 60 °C for 1 h and subsequently alkylated with 10 mmol l^−1^ iodoacetamide at 37 °C for 30 min in the dark. The final urea concentration was decreased to 1 mol l^−1^ with 20 mmol l^−1^ aqueous ammonium bicarbonate solution to ensure the efficiency of trypsin digestion. Trypsin was added to the sample in a protease to protein ratio of 1:25 (*w*/w). After 16–18 h incubation at 37 °C, digestion was stopped with 1% (*v*/v) acetic acid. Peptides in the supernatant after centrifugation (10 min, 16,000 x g) were desalted and purified using μC18-ZipTip columns (Merck Millipore, Darmstadt, Germany). Elution buffer was evaporated in a vacuum centrifuge and peptides were dissolved in 20 μl 0.1% (v/v) aqueous acetic acid containing 2% (v/v) acetonitrile (ACN). Previously, we had tested digestion efficiency of this protocol for *E. coli* K12 cell pellets by 1D gel analysis with silver staining and revealed to be >99.9% [66]. Samples were stored short-term at −20 °C before shotgun MS or SRM analysis.

### Preparation and proteome analysis of 2D PAGE

In order to detect a possible phosphorylation of ArcA during Cpx activation on protein level, two dimensional polyacrylamide gel electrophoresis (2D PAGE) was conducted. Each 400 μg protein extracts (see above for preparation) of *E. coli* grown under WT conditions, gentamicin treatment or Cpx activation (*nlpE* overexpression) were utilized. In the first dimension, proteins were separated by their isoelectric point (pI) during isoelectric focusing on immobilized pH gradient (IPG) strips using a Multiphor™ II system (GE Healthcare Life Sciences, Freiburg, Germany) according to factory instructions in a pH range between 4.5 and 5.5 to ensure a high resolution in the expected range of ArcA (pI 5.2 according to ExPASy.org).

In the second dimension proteins were separated by their molecular mass. The IPG strips were first equilibrated for 15 min at room temperature in aqueous buffer consisting of 6 mol l^−1^ urea, 1.5 mol l^−1^ Tris HCl, pH 8.8, 87% (v/v) glycerol, 20% (m/v) sodium dodecyl sulfate (SDS) and 10 mg ml^−1^ dithiothreitol. Subsequently, a second equilibration took place for 15 min with a similar buffer only replacing dithiothreitol with 25 mg ml^−1^ iodoacetamide and a little bromophenol blue. Equilibrated IPG strips were transferred onto a 0.5% (m/v) agarose gel prepared in running buffer [72 g glycin, 15 g Tris, and 25 ml 20% (m/v) SDS filled up to 500 ml with *A. dest.*]. Proteins were separated at 20 mA in about 1.5 h. Coomassie Brilliant Blue G-250 (Merck Millipore, Darmstadt, Germany) staining allowed visualization of separated proteins using the program Delta 2D (DECODON, Greifwald, Germany) after scanning the gels. Spots from each two gels (WT and gentamicin treatment or WT and Cpx activation) were aligned allowing size comparison and determination of presence or absence in one of the two conditions.

For protein identification, selected single protein spots (Additional file 1: Figure S3) were cut out from the gel with the help of a pipette tip and transferred to a 1.7 ml reaction tube. To discolor the gel 200 μl 200 mmol l^−1^ aqueous ammonium bicarbonate were added to the gel spot and allowed for 15 min incubation at 37 °C. The supernatant was removed and the step was repeated until the dye was completely removed. Afterwards, the gel was dehydrated by adding 100 μl acetonitrile for 15 min at 37 °C. After removal of the supernatant, the dehydration was repeated once. To the dried gel piece 10 μl 10 ng μl^−1^ trypsin (Promega, Madison, WI, USA, to detect tryptic peptides cleaved after lysine and arginine) dissolved or 10 ng μl^−1^ proteinase K (Sigma-Aldrich, St. Louis, USA, to increase number of detected peptides due to less specific cleavage) in 20 mmol l^−1^ aqueous ammonium bicarbonate were added. After 1 h incubation at room temperature, excessive protease solution was removed, if the gel was not fully reconstituted with liquid, the required volume of 20 mmol l^−1^ aqueous ammonium bicarbonate was added to the sample. Subsequent tryptic digestion was allowed for 14 h at 37 °C in horizontal position of the reaction tubes to avoid drying the gel. Peptides were extracted from the gel first, in 10 μl 0.1% (*v*/v) aqueous acetic acid for 30 min in a ultrasonication bath and, second, after separation of the first supernatant, by adding 10 μL 50% (v/v) acetonitrile in 0.05% (v/v) aqueous acetic acid under same conditions. Both supernatant containing peptides were combined and freeze-dried by lyophilization. Dried peptides were reconstituted in 40 μl 0.1% (v/v) aqueous acetic acid and purified with μC18-ZipTip columns (Merck Millipore, Darmstadt, Germany) and prepared for nanoLC-MS/MS as described above.

### Data acquisition by mass spectrometry

Equal to our previously published study [34] for proteome profiling peptide separation was performed on a NanoAcquity BEH130 C18 column (10 cm length, 100 μM inner diameter and 1.7 μm particle size) using a nanoAcquity UPLC (Waters, Manchester, UK). Separated peptides were ionized using electrospray and analyzed with an LTQ Orbitrap Velos mass spectrometer (Thermo Electron, Bremen, Germany) operated in data-dependent mode. Up to 20 of the most intense ions were sequentially isolated for collision induced dissociation (CID) in the linear ion trap. Shotgun LC-MS/MS analysis was carried out for four or five [correlation study between CpxA or ArcA and other detected proteins under WT, Cpx activating conditions (*nlpE* overexpression) and gentamicin treatment of *E. coli* WT strain], Additional file 4: Table S3) or four (influence of gentamicin on the proteome of WT or *cpxAR* mutant, Additional file 2: Table S1**;** Additional file 3: Table S2) independent biological replicates (BR) per condition.

Protein digests from the 2D gel were investigated on a Q Exactive mass spectrometer (Thermo Fisher Scientific, Waltham, MA, USA) after separation of peptides using a Dionex UltiMate 3000 RSLC nano-LC system (Dionex/Thermo Fisher Scientific, Idstein, Germany), and ionization with a TriVersa NanoMate source (Advion, Ltd., Harlow, UK). Here, after the first analysis peptides were fragmented using higher energy collision dissociation (HCD) instead of CID. Further details on shotgun data acquisition are provided as Additional files.

For SRM analysis, a nano-HPLC (EASY-nLC, Proxeon Biosystems A/S, Odense, Denmark) with the help of an Acclaim PepMap 100 reverse phase column (3 μm, 75 μm i.d. × 150 mm, LC Packings, Dionex, Idstein, Germany) was coupled to a TSQ Vantage (Thermo Electron). First, separated and ionized targeted peptides (precursors) were analyzed in the first quadrupole. After fragmentation by CID the corresponding likewise targeted products were analyzed in a further quadrupole. Required collision energy (CE) was optimized at precursor level beginning from factory defaults (depending on the m/z ratio of the precursor) by applying different eV in steps of + or −2 eV. Settings for final SRM analyses are provided as Additional files. For each peptide, the doubly charged precursor and the four most abundant product ions (transitions) were chosen for SRM acquisition (Additional file 1: Table S4). SRM data were recorded for five independent biological replicates for each condition.

### Analysis of proteome data

Mass spectrometric data from proteome profiling was investigated with the Rosetta Elucidator software (Ceiba Solutions, Boston, MA, USA). Protein identifications resulted from an automated database search against a Swiss-Prot database rel. 06–2014 limited to *E. coli* K12 entries using Sequest v. 2.7. Quantitative analysis was based on summed intensities of aligned single isotope features representing peptides (PeptideTeller probability >0.95). Only proteins that were identified by at least two peptides or by one peptide provided that the sequence coverage exceeded 10%, respectively, were subjected for further analysis. With the help of the Genedata Analyst software v8.2 (Genedata AG, Basel, Switzerland) protein intensity values were median normalized and statistically analyzed using two group t-test together with multiple testing corrections after to Benjamini-Hochberg.

The normalized intensity values of each protein from the complete dataset of four conditions and five biological replicates (for *nlpE* overexpression only four biological replicates were available) were used to determine correlations between the intensity behavior of CpxA and ArcA and all other quantified proteins. Therefore, a linear model was applied in Genedata Analyst and statistically tested. Correlations comprising a q-value (Benjamini-Hochberg corrected) < 0.05 were regarded as statistically significant. Positive correlation values indicated similar, negative values an opposite behavior of protein intensities. Values >0.8 were defined significantly positively correlated and values < −0.8 as significantly negatively correlated. A value of 1 indicated the same behavior as the compared protein.

Ratios in comparison to the WT or between control and gentamicin treated sample (four BR) were calculated from the mean of all BRs per condition. Ratios between two conditions with values <0.5 or >2, along with a multiple testing corrected q-value <0.05 were regarded as regulated in the corresponding condition.

Data from separated proteins from 2D gels were identified using the MASCOT search algorithm against the *E. coli* database (limited to tryptic peptides or non-limited when proteinase K was used for hydrolysis of proteins) mentioned above allowing a false discovery rate of <1% and carbamidomethylation of cysteine set as static, and oxidation of methionine and phosphorylation of aspartate as variable modification.

For SRM analysis method development and optimization as well as quantification were accomplished using the open source program Skyline v2.5 [67]. The final transition list is provided as Additional file 1: Table S4. Quantification was performed as described recently [34]. Pairs of heavy and light peptides were identified by equal peak elution pattern and retention time. Dilution series of each heavy standard peptide (0, 0.1, 0.5, 1, 5, 10, 50, and 100 fmol μg^−1^ protein) mixed with background of WT bacteria were acquired as duplicates by SRM after tryptic digestion. From that the linear range (R^2^ > 0.99), in which absolute quantification was possible, was determined for each peptide. The peptide per protein with the highest intensity and thereby best signal to noise ratio was chosen for quantification. Finally, each of the samples was spiked with 0.5 fmol μg^−1^ or 10 fmol μg^−1^ for each peptide. The ratio from the peak area of the heavy (synthetic peptide) to light (natural sample peptide) eluting at same retention time, which amounted closer to one (single point calibration) was utilized for absolute quantification. Average values and coefficient of variance were calculated over all replicates for each condition.

## Additional files

Additional file 1: Supporting information and supporting figures. (DOCX 2129 kb)

Additional file 2: Table S1. Proteome profile of *E. coli* MG1655 and its isogenic *cpxAR* mutant with and without incubation with 5 μg ml^−1^ gentamicin. (XLSX 882 kb)

Additional file 3: Table S2. Proteins regulated upon gentamicin treatment. (XLSX 210 kb)

Additional file 4: Table S3. Correlation studies between intensities of CpxA or ArcA and other detected proteins including WT condition, Cpx induction, and gentamicin treatment. (XLSX 496 kb)

## Abbreviations

ArcA ~ P: Phosphorylated ArcA
BACTH: Bacterial two-hybrid
CpxR ~ P: Phosphorylated CpxR
SRM: Single reaction monitoring
TCS: Two - component system
