# Supplementary material for: The role of the two-component systems Cpx and Arc in protein alterations upon gentamicin treatment in Escherichia coli
Source: BMC Microbiol. 2017 Sep 18;17:197. doi: 10.1186/s12866-017-1100-9 (PMC5604497; doi:10.1186/s12866-017-1100-9)
Supplement: Supplementary file 1 — Supporting information and supporting figures. (DOCX 2129 kb) [file 12866_2017_1100_MOESM1_ESM.docx]

**Supporting Information**

**The role of the two-component systems Cpx and Arc in protein alterations upon gentamicin treatment in *Escherichia coli***

Emina Ćudić^1^*, Kristin Surmann^2^*, Gianna Panasia^1,3^, Elke Hammer^2^, Sabine Hunke^1#^

*These authors contributed equally to this work.

^#^corresponding author

^1^FB 5 Microbiology, Department of Biology/Chemistry, University Osnabrück, Barbarastraße 11, 49076 Osnabrück, Germany

^2^Department of Functional Genomics, Interfaculty Institute of Genetics and Functional Genomics, University Medicine Greifswald, Friedrich-Ludwig-Jahn-Straße 15A, 17475 Greifswald, Germany

^3^present address: Institute of Molecular Microbiology and Biotechnology, Department of Biology, Corrensstraße 3, 48149 Münster, Germany

**Correspondence:**

Dr. rer. nat. Sabine Hunke

E-mail: Sabine.Hunke@UOS.de

Tel.: +49 541 969-7141

Fax: +49 541 969-3942

**Overview**

**Details on data acquisition for shotgun proteomics and SRM.**

**Figure S1: Quantification of CpxA-Strep and ArcA-Snap band densities acquired by mSPINE-experiments.**

**Figure S2: Growth of *E. coli* K12-derivate strain MG1655 with and without the addition of 5 µg ml^-1^ gentamicin.**

**Figure S3: 2D PAGE of *E. coli* protein extract of WT conditions and after gentamicin treatment.**

**Table S1: Proteome profile of *E. coli* MG1655 and its isogenic *cpxAR* mutant with and without incubation with 5 µg ml^-1^ gentamicin.** Data from each four independent biological replicates per condition are presented. The file contains protein accession and general properties including functional categorization by gene ontology information on regulation by the Cpx system [1] or the Arc system [2]. Furthermore, median normalized intensities are given per replicate and as average values per condition with correlation of variation (CV) information. Ratios of protein intensities were calculated between all conditions and the wildtype (WT) and between gentamicin treated and untreated cells of the mutant. Significantly changed protein levels with ratios > 2 were labeled red or < 0.5 blue and p-values and q-values (multiple testing corrections) < 0.05 from a two group t-test in green. Remark: Since an alignment search was applied on MS1 level, peaks were assigned to CpxA and CpxR in the wildtype and the *cpxAR* mutant. For CpxA, the fold change between the WT and the mutant measured only 2. However, additional analysis on MS2 level (data not shown) and previous controls of the mutant [1] ensure that CpxA and was not produced in the mutant. The wrong assignments probably occurred since CpxA was detected only in very low amounts in only one sample of the WT with range 10^5^ close to detection limit. Nevertheless, for exact quantification alignment search is required. The data are provided as separate Excel file (**Additional file 2**).

**Table S2: Proteins regulated upon gentamicin treatment.** From **Table S1**, proteins with significantly changed proteins levels were sorted by increased (Tab1, induction) or decreased (Tab2, inhibition) levels in comparison to the wildtype (WT) and complemented with information on promotor regulations. Significantly altered protein levels were characterized by a multiple correction adjusted q-value < 0.05 and a ratio > 2 (induction, red) or < 0.5 (inhibition, blue). The q-values of selected proteins showing a trend of induction but no significant regulation (q < 0.05, 1.5 < ratio < 2) were highlighted in dark red. The data are provided as separate Excel file (**Additional file 3**).

**Table S3: Correlation studies between intensities of CpxA or ArcA and other detected proteins including WT condition, Cpx induction, and gentamicin treatment.** Correlations among normalized protein intensities were calculated in a linear model over three different conditions and in total 14 samples. The table contains median normalized intensity values for each replicate and correlation coefficients and p-value and Benjamini-Hochberg corrected q-values calculated for CpxA and ArcA among each sample. Significantly correlating values are characterized by p- and q-values < 0.05 (green) and correlation coefficients > 0.8 (red) or < -0.8 (blue). The data are provided as separate Excel file (**Additional file 4**).

**Table S4: Transitions for identification and quantification of Arc and Cpx proteins by single reaction monitoring.**

**Details on data acquisition for shotgun MS and SRM**

**Details on data acquisition for proteomics profiling by shotgun proteomics**

| **LC-Parameters** | **Settings** |
| --- | --- |
| Instrument | UPLC (Waters, Manchester, UK) |
| LC-column | Acclaim PepMap 100 reverse phase column (3 *µ*m, 75 *µ*m i.d x 150 mm, LC Packings, Dionex, Idstein, Germany) |
| LC-gradient | 0 min-1%ACN-35-1-36-5-245-25-305-60-306-99-310-1-320-1 |
| Solvent flow rate | 300 nL/min |
| **MS-Parameters** | **Settings** |
| Instrument | Orbitrap Velos (Thermo Fisher Scientific, Waltham, MA, USA) |
| Mass range | m/z 300-2,000 |
| Resolution | 60,000 at m/z 400 |
| Name of peaklist-generating software and release version (number or date) | ReadW in Sorcerer built 4.04 (SageN Research Inc., Milpitas, CA, USA) with default parameters |
| Name of the search engine and release version (number or date) | Sequest (v. 2.7) in Sorcerer built 4.04 (SageN) |
| Enzyme specificity considered | Fully tryptic |
| # of missed cleavages permitted | Missed cleavages=0 |
| Fixed modification(s) (including residue specificity) | Carbamidomethylation at cysteine |
| Variable modification(s) (including residue specificity) | Oxidation on methionine |
| Mass tolerance for precursor ions | 10 ppm |
| Mass tolerance for fragment ions | 1 Da |
| Name of database searched and release version/date | Swiss-Prot database rel. 06_2014 limited to *E. coli* K12 entries |
| Threshold score/E-value for accepting *individual* MS/MS Spectra | Peptide Teller false positive rate 1% |
| Software/method used to evaluate site assignment | No PTM reported |

**Presentation of protein identification results**

| **Information requested** | **Reported** |
| --- | --- |
| Accession number | UniprotAccession |
| Number of *unique* (in terms of amino acid sequence) peptides identified, % sequence coverage identified from MS/MS data, protein’s name, function, MW, pI, score, peptide sequences, etc. | Table S2 |

**Details on data acquisition for protein identification from isolated 2D PAGE spots**

| **LC-Parameters** | **Settings** |
| --- | --- |
| Instrument | Ultimate 3000 RSLC (Thermo Fisher Scientific) |
| Trap column | 75 μm inner diameter, packed with 3 μm C18 particles, 100 Å pore size (Acclaim PepMap100, Thermo Scientific) |
| Analytical column | Accucore 150-C18, (Thermo Fisher Scientific)  25 cm x 75 μm, 2,6 μm C18 particles, 150 Å pore size |
| Buffer system | Binary buffer system consisting of 0.1% acetic acid, 5% ACN (buffer A) and 100% ACN in 0.1% acetic acid (buffer B) |
| LC-gradient | linear gradient of buffer B from 2% up to 25% in 120 min |
| Solvent flow rate | 300 nL/min |
| **MS-Parameters** | **Settings** |
| Instrument | Q Exactive mass spectrometer (Thermo Scientific) |
| Mass range | m/z 300-1,650 |
| Resolution | 70,000 |
| Name of the search engine and release version (number or date) | Mascot v2.5.1 |
| Enzyme specificity considered | Fully tryptic or non-specific (proteinase K) |
| # of missed cleavages permitted | Missed cleavages=0 |
| Fixed modification(s) (including residue specificity) | Carbamidomethylation at cysteine |
| Variable modification(s) (including residue specificity) | Oxidation on methionine  Phosphorylation on aspartate |
| Mass tolerance for precursor ions | 10 ppm |
| Mass tolerance for fragment ions | 0.02 Da |
| Name of database searched and release version/date | Swiss-Prot database rel. 06_2014 limited to *E. coli* K12 entries |
| Threshold score/E-value for accepting *individual* MS/MS Spectra | False discovery rate < 1 % |

**Details on data acquisition for SRM**

| **LC-Parameters** | **Settings** |
| --- | --- |
| LC gradient | 0 min-5%ACN-3-5-26-35-29-45-31-100-33-100-36-0 |
| Solvent flow rate | 300 nL/min |
| **MS-Parameters** | **Settings** |
| Resolution | MS1 R=0.7 full width at half maximum (FWHM), MS2 R= 2.5 FWHM |
| Dwell time | 20 ms per transition |
| Cycle time | 2.4 s/cycle |

SRM transitions are provided in Supporting Information Table S4. Results from absolute quantification are presented in Figure 4 and Table 1 in the manuscript.

**
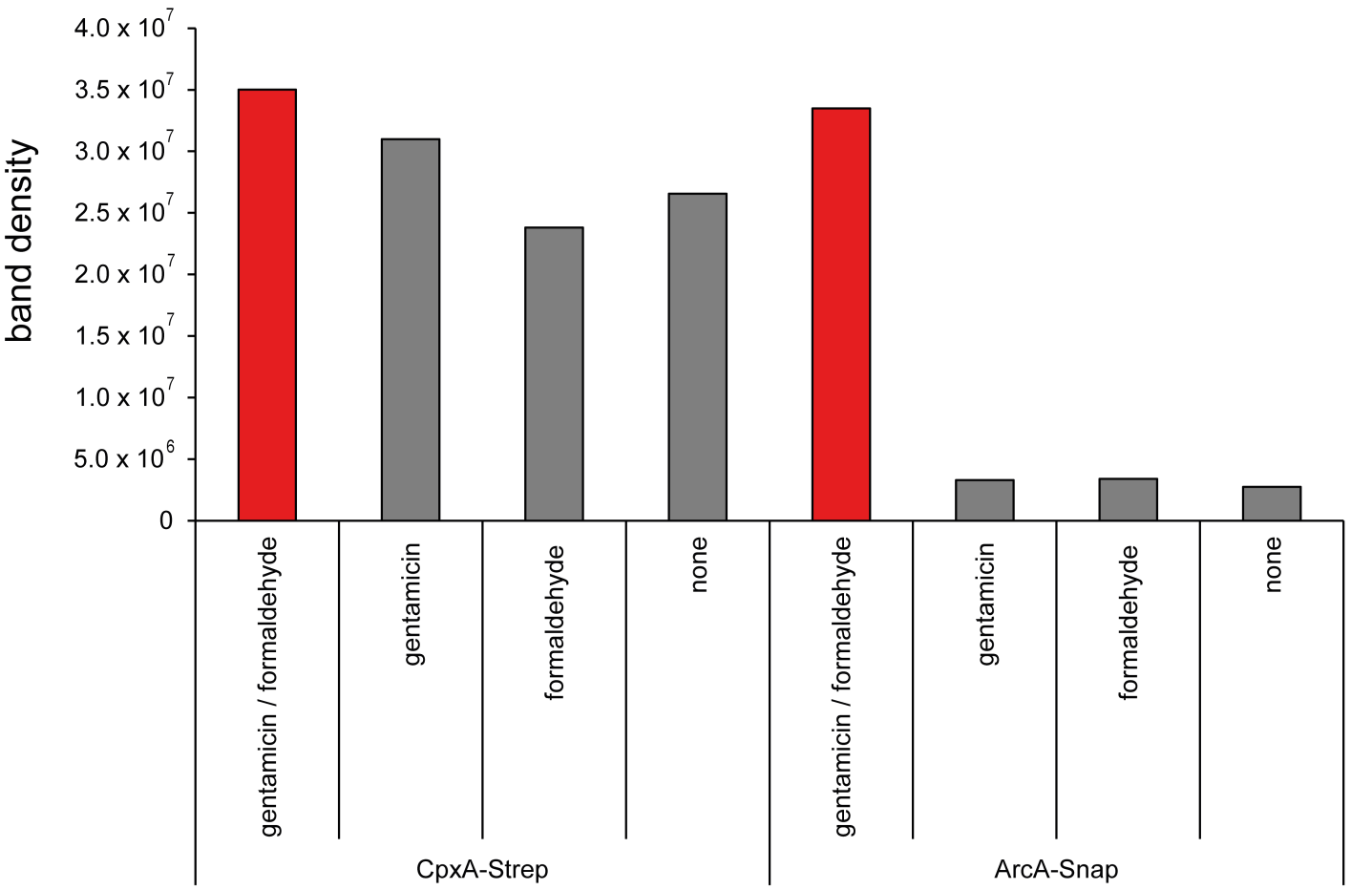
**

**Figure S1: Quantification of CpxA-Strep and ArcA-Snap band densities acquired by mSPINE-experiments.** The interaction between CpxA-Strep and ArcA-Snap was monitored by mSPINE. The respective immunoblot was employed for quantification of CpxA-Strep and ArcA-Snap band densities by ImageJ among all conditions tested. These comprise addition of gentamicin and formaldehyde (red bars), addition of gentamicin or formaldehyde only (grey bars), or upon absence of both (‘none’; grey bars).

**
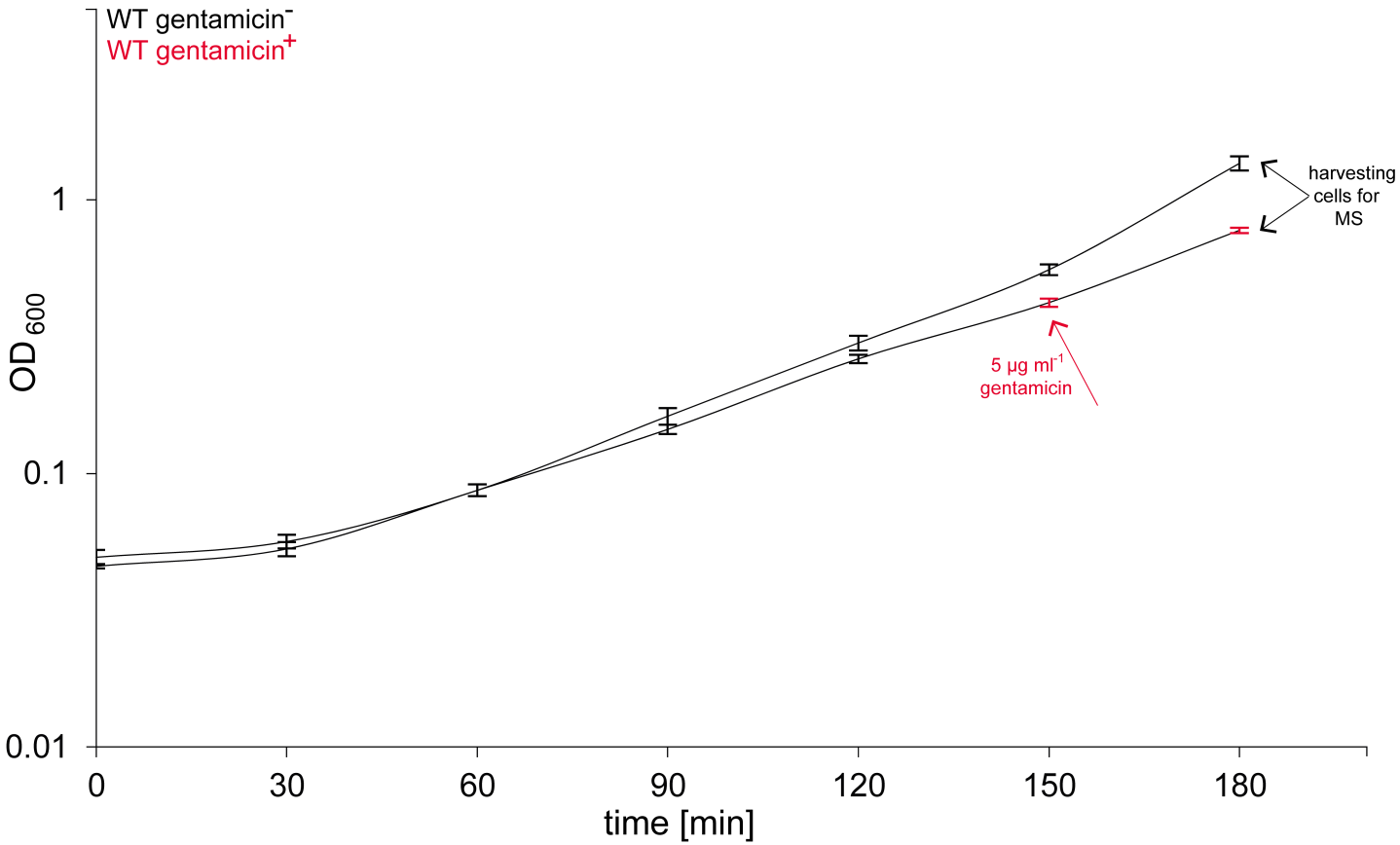
**

**Figure S2: Growth of *E. coli* K12-derivate strain MG1655 with and without the addition of 5 µg ml^-1^ gentamicin.** MG1655-cells were grown in LB-media for 2.5 h. The addition of 5 µg ml^-1^ gentamicin is indicated by the red arrow. The incubation was continued for additional 30 min prior to harvesting the cells for proteomic analyses indicated by the black arrows. The growth curves depict a mean of five biological replicates and standard deviations, respectively. The y-axis is formatted in log_10_ logarithmic scale.

**Figure S3: 2D PAGE of *E. coli* protein extract of WT conditions and after gentamicin treatment.** Each 400 µg protein from *E. coli* MG1655 grown under WT conditions (red) or gentamicin treatment (green) were separated by 2D PAGE and analyzed using the program Delta 2D (DECODON, Greifswald, Germany). After alignment of both gels the spots likely to contain ArcA from pre-experiments (data not shown) were detected in the overlay, cut, prepared for proteome analysis and measured by nanoLC-MS/MS. Two spots only differing in pH value (02 and 03 on both gels) and similar in abundance (yellow overlay) were identified as ArcA.

**Table S4: Transitions for identification and quantification of Arc and Cpx proteins by single reaction monitoring.**

**Arc proteins**

| **protein** | **peptide** | **precursor m/z** | **product m/z** | **optimized collision energy** | **heavy standard** | **detected in shotgun MS** | **R^2^ value for standard curve of heavy peptide** | **minimum detectable concentration [fmol/µg protein]** |
| --- | --- | --- | --- | --- | --- | --- | --- | --- |
| ArcA | FNGWELDINSR | 675.825 | 604.305 | 24 | no | yes | 0.9994 | 0.5 |
|  |  | 675.825 | 717.389 | 24 | no | yes |  |  |
|  |  | 675.825 | 846.432 | 24 | no | yes |  |  |
|  |  | 675.825 | 1089.532 | 24 | no | yes |  |  |
|  |  | 680.83 | 614.313 | 24 | yes | yes |  |  |
|  |  | 680.83 | 727.397 | 24 | yes | yes |  |  |
|  |  | 680.83 | 856.44 | 24 | yes | yes |  |  |
|  |  | 680.83 | 1099.541 | 24 | yes | yes |  |  |
|  | **SLIGPDGEQYK** | **603.804** | **624.299** | **21** | **no** | **no** | **1** | **0.1** |
|  |  | **603.804** | **739.326** | **21** | **no** | **no** |  |  |
|  |  | **603.804** | **836.378** | **21** | **no** | **no** |  |  |
|  |  | **603.804** | **893.4** | **21** | **no** | **no** |  |  |
|  |  | **607.811** | **632.313** | **21** | **yes** | **no** |  |  |
|  |  | **607.811** | **747.34** | **21** | **yes** | **no** |  |  |
|  |  | **607.811** | **844.393** | **21** | **yes** | **no** |  |  |
|  |  | **607.811** | **901.414** | **21** | **yes** | **no** |  |  |
| ArcB | **DNIAQLNQEIAVR** | **742.397** | **829.453** | **22** | **no** | **no** | **0.999** | **0.1** |
|  |  | **742.397** | **942.537** | **22** | **no** | **no** |  |  |
|  |  | **742.397** | **1070.595** | **22** | **no** | **no** |  |  |
|  |  | **742.397** | **1141.632** | **22** | **no** | **no** |  |  |
|  |  | **747.401** | **839.461** | **22** | **yes** | **no** |  |  |
|  |  | **747.401** | **952.545** | **22** | **yes** | **no** |  |  |
|  |  | **747.401** | **1080.604** | **22** | **yes** | **no** |  |  |
|  |  | **747.401** | **1151.641** | **22** | **yes** | **no** |  |  |
|  | FTQQGQVTVR | 582.312 | 602.362 | 22 | no | no | 0.9992 | 0.1 |
|  |  | 582.312 | 659.383 | 22 | no | no |  |  |
|  |  | 582.312 | 787.442 | 22 | no | no |  |  |
|  |  | 582.312 | 915.501 | 22 | no | no |  |  |
|  |  | 587.316 | 612.37 | 22 | yes | no |  |  |
|  |  | 587.316 | 669.392 | 22 | yes | no |  |  |
|  |  | 587.316 | 797.45 | 22 | yes | no |  |  |
|  |  | 587.316 | 925.509 | 22 | yes | no |  |  |
|  | SFLDASPDLVFYR | 765.385 | 909.483 | 23 | no | yes | 0.9988 | 0.5 |
|  |  | 765.385 | 996.515 | 23 | no | yes |  |  |
|  |  | 765.385 | 1067.552 | 23 | no | yes |  |  |
|  |  | 765.385 | 1182.579 | 23 | no | yes |  |  |
|  |  | 770.39 | 919.491 | 23 | yes | yes |  |  |
|  |  | 770.39 | 1006.523 | 23 | yes | yes |  |  |
|  |  | 770.39 | 1077.56 | 23 | yes | yes |  |  |
|  |  | 770.39 | 1192.587 | 23 | yes | yes |  |  |

**Cpx proteins**

| **protein** | **peptide** | **precursor m/z** | **product m/z** | **optimized collision energy** | **heavy standard** | **detected in shotgun MS** | **R^2^ value for standard curve of heavy peptide** | **minimum detectable concentration [fmol/µg protein]** |
| --- | --- | --- | --- | --- | --- | --- | --- | --- |
| CpxA | **AEDSPLGGLR** | **507.764** | **402.246** | **19** | **no** | **no** | **0.9994** | **0.5** |
|  |  | **507.764** | **612.383** | **19** | **no** | **no** |  |  |
|  |  | **507.764** | **699.415** | **19** | **no** | **no** |  |  |
|  |  | **507.764** | **814.442** | **19** | **no** | **no** |  |  |
|  |  | **512.768** | **412.254** | **19** | **yes** | **no** |  |  |
|  |  | **512.768** | **622.391** | **19** | **yes** | **no** |  |  |
|  |  | **512.768** | **709.423** | **19** | **yes** | **no** |  |  |
|  |  | **512.768** | **824.45** | **19** | **yes** | **no** |  |  |
|  | LLLVTTEGR | 501.303 | 462.231 | 19 | no | yes | 0.9987 | 0.1 |
|  |  | 501.303 | 563.278 | 19 | no | yes |  |  |
|  |  | 501.303 | 662.347 | 19 | no | yes |  |  |
|  |  | 501.303 | 775.431 | 19 | no | yes |  |  |
|  |  | 506.307 | 472.239 | 19 | yes | yes |  |  |
|  |  | 506.307 | 573.287 | 19 | yes | yes |  |  |
|  |  | 506.307 | 672.355 | 19 | yes | yes |  |  |
|  |  | 506.307 | 785.439 | 19 | yes | yes |  |  |
| CpxP | **DVTQWQK** | **452.73** | **461.251** | **18** | **no** | **no** | **0.9998** | **0.5** |
|  |  | **452.73** | **589.309** | **18** | **no** | **no** |  |  |
|  |  | **452.73** | **690.357** | **18** | **no** | **no** |  |  |
|  |  | **452.73** | **789.425** | **18** | **no** | **no** |  |  |
|  |  | **456.737** | **469.265** | **18** | **yes** | **no** |  |  |
|  |  | **456.737** | **597.323** | **18** | **yes** | **no** |  |  |
|  |  | **456.737** | **698.371** | **18** | **yes** | **no** |  |  |
|  |  | **456.737** | **797.44** | **18** | **yes** | **no** |  |  |
|  | LLTPEQQAVLNEK | 741.912 | 503.282 | 22 | no | yes | 0.9997 | 0.1 |
|  |  | 741.912 | 673.388 | 22 | no | yes |  |  |
|  |  | 741.912 | 1155.6 | 22 | no | yes |  |  |
|  |  | 741.912 | 1256.648 | 22 | no | yes |  |  |
|  |  | 745.919 | 511.297 | 22 | yes | yes |  |  |
|  |  | 745.919 | 681.402 | 22 | yes | yes |  |  |
|  |  | 745.919 | 1163.615 | 22 | yes | yes |  |  |
|  |  | 745.919 | 1264.662 | 22 | yes | yes |  |  |
|  | LVTAENFDENAVR | 739.368 | 850.405 | 22 | no | yes | 0.9997 | 0.1 |
|  |  | 739.368 | 964.448 | 22 | no | yes |  |  |
|  |  | 739.368 | 1093.491 | 22 | no | yes |  |  |
|  |  | 739.368 | 1265.576 | 22 | no | yes |  |  |
|  |  | 744.372 | 860.414 | 22 | yes | yes |  |  |
|  |  | 744.372 | 974.457 | 22 | yes | yes |  |  |
|  |  | 744.372 | 1103.499 | 22 | yes | yes |  |  |
|  |  | 744.372 | 1275.584 | 22 | yes | yes |  |  |
| CpxR | **EHLSQEVLGK** | **570.306** | **545.329** | **22** | **no** | **yes** | **0.9997** | **0.5** |
|  |  | **570.306** | **673.388** | **22** | **no** | **yes** |  |  |
|  |  | **570.306** | **760.42** | **22** | **no** | **yes** |  |  |
|  |  | **570.306** | **873.504** | **22** | **no** | **yes** |  |  |
|  |  | **574.313** | **553.344** | **22** | **yes** | **yes** |  |  |
|  |  | **574.313** | **681.402** | **22** | **yes** | **yes** |  |  |
|  |  | **574.313** | **768.434** | **22** | **yes** | **yes** |  |  |
|  |  | **574.313** | **881.518** | **22** | **yes** | **yes** |  |  |
|  | GSELDR | 676.326 | 403.23 | 26 | no | no | not applicable | not applicable |
|  |  | 676.326 | 502.214 | 26 | no | no |  |  |
|  |  | 676.326 | 532.273 | 26 | no | no |  |  |
|  |  | 676.326 | 619.305 | 26 | no | no |  |  |
|  |  | 686.334 | 413.238 | 26 | yes | no |  |  |
|  |  | 686.334 | 502.214 | 26 | yes | no |  |  |
|  |  | 686.334 | 542.281 | 26 | yes | no |  |  |
|  |  | 686.334 | 629.313 | 26 | yes | no |  |  |

For each protein of the Cpx and Arc system, proteotypic peptides, m/z ratios of heavy and light precursors and products are listed as well as the optimized collision energy on protein levels. Peptides that were detected in an earlier approach [1] also by shotgun MS analysis are labelled “yes”, peptides which were derived from theoretical digestion with “no”. Furthermore, the R^2^ values of standard curves (two technical replicates, representing the linear range for absolute quantification) for each heavy standard peptide are presented. For each protein, the peptide with the highest R^2^ value was labelled in bold letters and used for further quantification of protein level. The Cpx proteins were quantified previously [1] and now used for comparison with the Arc system.

**References**

1. Surmann K, Ćudić E, Hammer E, Hunke S: **Molecular and proteome analyses highlight the importance of the Cpx envelope stress system for acid stress and cell wall stability in *Escherichia coli*.** *Microbiologyopen* 2016, **5(**4):582-596.
2. Kohanski MA, Dwyer DJ, Wierzbowski J, Cottarel G, Collins JJ: **Mistranslation of membrane proteins and two-component system activation trigger antibiotic-mediated cell death.** *Cell* 2008, **135**(4):679-690.
